# Spermine synthase and MYC cooperate to maintain colorectal cancer cell survival by repressing Bim expression

**DOI:** 10.1038/s41467-020-17067-x

**Published:** 2020-06-26

**Authors:** Yubin Guo, Qing Ye, Pan Deng, Yanan Cao, Daheng He, Zhaohe Zhou, Chi Wang, Yekaterina Y. Zaytseva, Charles E. Schwartz, Eun Y. Lee, B. Mark Evers, Andrew J. Morris, Side Liu, Qing-Bai She

**Affiliations:** 1 Guangdong Provincial Key Laboratory of Gastroenterology, Department of Gastroenterology, Nanfang Hospital, Southern Medical University, 510515 Guangzhou, China; 2 Markey Cancer Center, University of Kentucky College of Medicine, Lexington, KY 40506 USA; 3 Department of Pharmacology and Nutritional Sciences, University of Kentucky College of Medicine, Lexington, KY 40506 USA; 4 Superfund Research Center, University of Kentucky, Lexington, KY 40536 USA; 5 Department of Biostatistics, University of Kentucky College of Public Health, Lexington, KY 40506 USA; 6 Department of Toxicology and Cancer Biology, University of Kentucky College of Medicine, Lexington, KY 40506 USA; 7 The Greenwood Genetic Center, Greenwood, SC 29646 USA; 8 Department of Pathology and Laboratory Medicine, University of Kentucky College of Medicine, Lexington, KY 40506 USA; 9 Department of Surgery, University of Kentucky College of Medicine, Lexington, KY 40506 USA; 10 Division of Cardiovascular Medicine and the Gill Heart Institute, University of Kentucky College of Medicine, and Lexington Veterans Affairs Medical Center, Lexington, KY 40506 USA

**Keywords:** Cancer metabolism, Colorectal cancer

## Abstract

Dysregulation of polyamine metabolism has been linked to the development of colorectal cancer (CRC), but the underlying mechanism is incompletely characterized. Here, we report that spermine synthase (SMS), a polyamine biosynthetic enzyme, is overexpressed in CRC. Targeted disruption of *SMS* in CRC cells results in spermidine accumulation, which inhibits FOXO3a acetylation and allows subsequent translocation to the nucleus to transcriptionally induce expression of the proapoptotic protein Bim. However, this induction is blunted by MYC-driven expression of miR-19a and miR-19b that repress Bim production. Pharmacological or genetic inhibition of MYC activity in SMS-depleted CRC cells dramatically induces Bim expression and apoptosis and causes tumor regression, but these effects are profoundly attenuated by silencing *Bim*. These findings uncover a key survival signal in CRC through convergent repression of Bim expression by distinct SMS- and MYC-mediated signaling pathways. Thus, combined inhibition of SMS and MYC signaling may be an effective therapy for CRC.

## Abstract (web summary)

Polyamine metabolism is frequently dysregulated in cancers. Here, the authors show that a polyamine biosynthetic enzyme, spermine synthase, is overexpressed in colorectal cancers and cooperates with MYC to prevent cancer cell apoptosis by repression of proapoptotic protein, Bim.

## Introduction

**Fig. 1:**
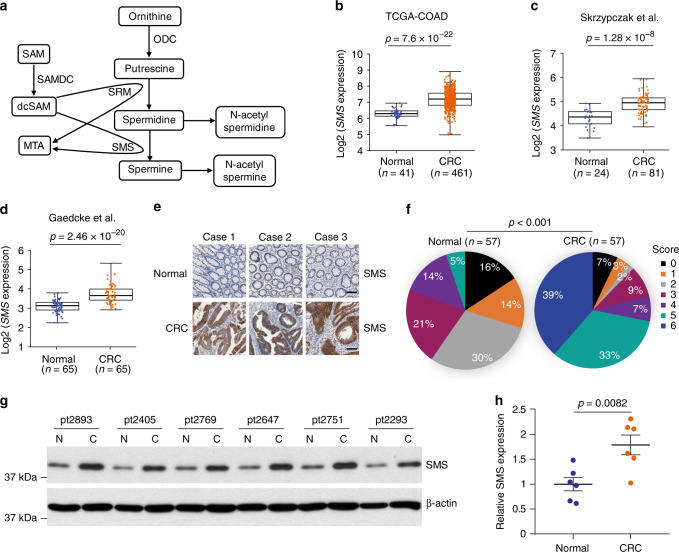
SMS is overexpressed in CRC. **a** Schematics of polyamine metabolism pathway. ODC ornithine decarboxylase, SRM spermidine synthase, SMS spermine synthase, SAMDC *S*-adenosylmethionine decarboxylase, SAM *S*-adenosylmethionine, dcSAM decarboxylated *S*-adenosylmethionine, MTA 5′-methylthioadenosine. **b–d** The mRNA expression levels of *SMS* were analyzed from three different datasets of human CRC specimens. Statistical significance between CRC tissues and normal controls was determined by the linear mixed model in **b** and **d**, or two-tailed two-sample *t* test in **c**, with the *p*-values indicated. **e**, **f** A tissue microarray containing 57 pairs of human CRC tumor and adjacent normal control tissues was subjected to IHC staining for SMS. Representative staining images are shown in **e**, and the percentages of the immunoreactivity scores are summarized in a diagram (**f**). Statistical significance was determined by the *χ*^2^-test with the *p*-value indicated. Scale bar, 100 µm. **g**, **h** Matched normal and tumor tissues from six patients with CRC were analyzed for SMS expression using western blot analysis (**g**). The expression of SMS protein was quantified by normalizing to β-actin using Image J. The relative SMS levels in tumor tissues (**h**) were compared with that of normal tissues within the same patient. The data are presented as mean values ±SEM (*n* = 6 biologically independent samples). The *p*-value by two-tailed unpaired *t* test is indicated. Source data are provided as a Source Data file.

Polyamines, including putrescine, spermidine, and spermine, are small polycationic metabolites that play a central role in cell growth^1,2^. Ornithine decarboxylase (ODC) encoded by *ODC1* gene is the rate-limiting step in polyamine biosynthesis and produces putrescine from ornithine^1^. Subsequently, putrescine is converted to spermidine and then spermine by two specific aminopropyltransferases, spermidine synthase (SRM), and spermine synthase (SMS), respectively (Fig. 1a). The aminopropyl donor for these reactions is decarboxylated *S*-adenosylmethionine (dcSAM), produced by *S*-adenosylmethionine decarboxylase (SAMDC) encoded by *AMD1* gene. The intracellular concentrations of polyamines are maintained within a narrow range through regulation of de novo synthesis, catabolism, and transportation. Alterations in polyamine levels have been associated with a variety of diseases, including neurodegeneration and cancer^1,3^. Mutations in human *SMS* have been found to cause the X-linked intellectual disability Synder-Robinson syndrome (SRS) with the pathological spermidine accumulation in SRS patients^4–6^. In cancer, polyamine metabolism is frequently dysregulated primarily through upregulation of the polyamine biosynthetic enzymes, which leads to elevated polyamine levels that are necessary for malignant transformation and tumor progression^1,2^. Thus, the polyamine metabolic pathway is an attractive target for anticancer therapies.

Numerous inhibitors that target polyamine biosynthetic and catabolic enzymes as well as polyamine transport have been developed and tested in preclinical and clinical studies^1^. Despite the early promise in vitro and several preclinical models of cancer, clinical trials using single agents targeting the polyamine pathway have generally proven to be disappointing^1^, except for difluoromethylornithine (DFMO), an irreversible inhibitor of ODC, which shows a single notable success in the treatment of anaplastic gliomas^7^. DFMO exhibits encouraging efficacy in ongoing clinical trials for neuroblastoma^8^ and prevention of colorectal cancer (CRC) by combination with sulindac^9^. Nevertheless, DFMO treatment is generally thought to exert a cytostatic, rather than a cytotoxic, effect, mainly due to the activation of compensatory mechanisms that result in increased polyamine transport or upregulation of SAMDC^1,10^.

Polyamine biosynthesis is promoted by multiple oncogenic signaling pathways derived from many prevalent mutations in cancers, including RAS, PI3K, and MYC^2^. These oncogenic events frequently occur in CRC^11^. Recent studies on metabolic changes and gene expression in CRC identify polyamines as among the most altered metabolic pathways and reveals an association between polyamine synthesis and tumorigenesis^12,13^. Polyamine synthesis is heavily regulated by the pluripotent transcription factor MYC at multiple levels. MYC increases expression of many polyamine synthesis genes, including *ODC1*, *SRM*, and *AMD1*, resulting an increase in the level of spermidine^14–16^. Spermidine has been shown to be important for MYC expression, cell proliferation, and malignant transformation^17,18^. However, in contrast to the carcinogenic properties of polyamines, excessive accumulation of polyamines including spermidine can be cytotoxic independent of their oxidative metabolites and facilitate the process of apoptosis in response to death stimuli^19,20^. Together, these studies indicate an important interplay between polyamine synthesis and oncogenic signaling. How cancer cells maintain a relatively high level of polyamines but below the toxic threshold to facilitate tumorigenesis is not well understood.

In this study, we report that SMS is highly expressed in CRC. Disruption of the *SMS* gene in CRC cells alters polyamine metabolism by dramatically reducing the levels of spermine and putrescine but producing excessive levels of spermidine. Our mechanistic studies indicate that overexpression of SMS is required for balancing spermidine levels to facilitate CRC cell growth. Furthermore, our work demonstrates that SMS cooperates with MYC to maintain CRC cell survival via distinct pathways that converge to repress expression of the proapoptotic protein Bim. Combined inhibition of SMS and MYC signaling induces synergistic apoptosis and tumor regression. This is, therefore, a promising strategy for CRC therapy.

## Results

### SMS is overexpressed in CRC

To determine if SMS expression is altered in CRC patients, we performed bioinformatic analysis of three different microarray datasets from human CRC specimens. *SMS* gene expression level was significantly increased in CRC specimens as compared with normal controls (Fig. 1b–d). Interestingly, additional analysis of *SMS* expression in patients with stage I–IV CRC revealed that *SMS* expression was upregulated upon tumor initiation when compared with normal controls, but no further increase of *SMS* expression was observed as tumors progressed through stage II–IV (Supplementary Fig. 1a). These data suggest that the increase of *SMS* expression is an early event in tumorigenesis. In addition, quantitative results obtained from immunohistochemical (IHC) staining of a tissue microarray with 57 pairs of human CRC tumors and adjacent normal control tissues also showed that SMS was overexpressed in CRC (Fig. 1e, f), which even occurred at the early stage of CRC (Supplementary Fig. 1b). Furthermore, we analyzed SMS protein expression in matched normal and tumor tissues obtained from six patients with CRC using western blot analysis. Consistent with IHC staining results, SMS protein levels were elevated by almost twofold in tumor tissues compared with normal controls (Fig. 1g, h; Supplementary Fig. 1c). Collectively, these data provide evidence that upregulation of SMS is associated with and may be required for CRC tumorigenesis.

### SMS deletion inhibits CRC cell growth

**Fig. 2:**
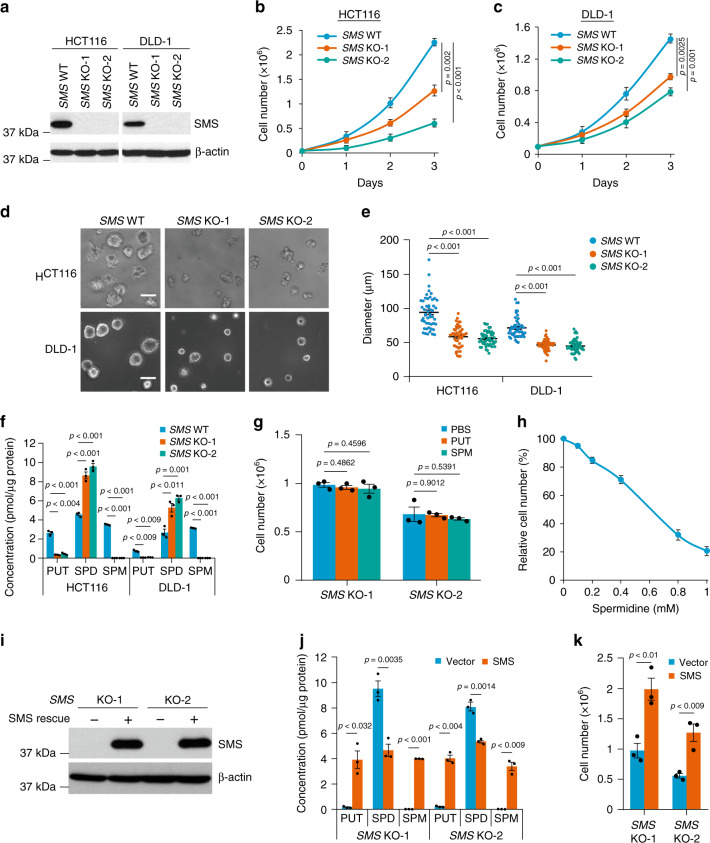
*SMS* knockout inhibits CRC cell growth associated with spermidine accumulation. **a** Cells from two *SMS* knockout (KO) HCT116 or DLD-1 clones, and their control wild-type (WT) cells were analyzed by western blot for the indicated proteins. **b**, **c** *SMS*-WT or *SMS*-KO HCT116 (**b**) and DLD-1 (**c**) cells were assessed for cell growth over 3 days. **d** Representative phase-contrast images of *SMS*-WT or *SMS*-KO HCT116 and DLD-1 cells cultured in 3D Matrigel for 4 days. Scale bar, 100 µm. **e** The diameter of 50 randomly chosen spheroids as shown in **d** were measured. **f** The levels of putrescine (PUT), spermidine (SPD), and spermine (SPM) in *SMS*-WT or *SMS*-KO HCT116 and DLD-1 cells were determined by LC-MS. **g** *SMS*-KO HCT116 cells were treated with 1 mM putrescine or 20 µM spermine for 3 days, following by counting the number of viable cells. **h** HCT116 cells treated with the indicated concentrations of spermidine were assessed for cell growth over 3 days. Results are expressed as a percentage of cell number relative to values obtained in the control cells. **i**, **j** *SMS*-KO HCT116 cells with re-expression of SMS or vector control were analyzed by western blot (**i**) for the indicated proteins, or by LC-MS analysis (**j**) for the levels of PUT, SPD, and SPM. **k** *SMS*-KO HCT116 cells with re-expression of SMS or vector control were assessed for cell growth over 3 days. All graphic data are presented as mean values ±SEM (*n* = 3 independent experiments in **b**, **c**, **e**–**h**, **j** and **k**; *n* = 50 spheroids in **d**). The indicated *p*-values were determined by two-tailed unpaired *t* test. Source data are provided as a Source Data file.

To define the role of SMS in CRC tumorigenesis, we first used short hairpin RNAs (shRNAs) to stably knock down SMS expression in HCT116 CRC cells (Supplementary Fig. 2a). Silencing *SMS* significantly inhibited HCT116 cell growth in both 2D and 3D cell culture conditions (Supplementary Fig. 2b–d). To further validate the role of SMS in CRC cell growth, we generated *SMS* knockout (KO) HCT116 and DLD-1 CRC cell lines using the CRISPR-Cas9 system^21^. Sequencing results confirmed that four types of frameshift indels were created in the targeted region of *SMS* exon 5 in these KO cells, but not in the wild-type (WT) cells (Supplementary Fig. 3a). Consistent with shRNA-mediated SMS knockdown, disruption of *SMS* also markedly inhibited the growth of HCT116 and DLD-1 cells from two different *SMS*-KO clones in both 2D and 3D cell culture conditions (Fig. 2a–e). Similar results were observed in three additional *SMS*-KO HCT116 or DLD-1 cell clones compared with their three additional *SMS-*WT cell clones (Supplementary Fig. 3b–d). To determine whether inhibition of CRC cell growth by disruption of *SMS* is mediated by an alteration in polyamine biosynthesis, cellular polyamine levels were measured by liquid chromatography mass spectrometry (LC-MS). Spermine levels were not detectable in both *SMS*-KO HCT116 and *SMS*-KO DLD-1 cells, and levels of the upstream polyamine putrescine were also drastically decreased (Fig. 2f). In contrast, spermidine levels were increased by more than twofold in *SMS*-KO cells compared with *SMS*-WT cells (Fig. 2f), while the levels of *N*^1^-acetylspermidine (Fig. 1a), the limiting derivative in polyamine catabolism^22^, were unaffected (Supplementary Fig. 4a). Since both SRM and SMS use dcSAM as a common aminopropyl donor to produce spermidine from putrescine and spermine from spermidine, respectively (Fig. 1a), the activities of SRM and SMS are limited by the availability of dcSAM and hence, the activity of SAMDC^23^. Similar to the reported increase in the activity and/or expression of SAMDC by *SMS* loss-of-function in several models^24,25^, a profound increase in SAMDC expression was observed in both *SMS*-KO HCT116 and *SMS*-KO DLD-1 cells (Supplementary Fig. 4b). Thus, the observed spermidine accumulation and putrescine reduction in *SMS*-KO CRC cells is likely attributed to the increased availability of dcSAM by SAMDC for spermidine biosynthesis in the absence of SMS activity. Notably, exogenously addition of putrescine and spermine in *SMS*-KO HCT116 cells to the levels similar to those detected in *SMS*-WT HCT116 cells did not alter spermidine accumulation or rescue cell growth inhibition by *SMS* deletion in both 2D and 3D cell culture conditions (Fig. 2f, g; Supplementary Fig. 4c–e), excluding a significant involvement of these polyamines in the *SMS*-KO cell growth. By contrast, addition of exogenous spermidine to the *SMS*-WT HCT116 cells resulted in a concentration-dependent growth inhibition associated with a correlated increase in the intracellular levels of spermidine (Fig. 2h; Supplementary Fig. 4f). Furthermore, re-expression of SMS in the *SMS*-KO HCT116 cells, which resulted in decreased spermidine level and restoration of putrescine and spermine to the level similar to those observed in the *SMS*-WT cells, could significantly reverse cell growth inhibition by *SMS* deletion (Fig. 2f, i–k). Taken together, these data indicate that *SMS* deletion-induced CRC cell growth inhibition is mainly attributed to the accumulation of spermidine.

### SMS deletion sensitizes CRC cells to MYC repression by JQ1

**Fig. 3:**
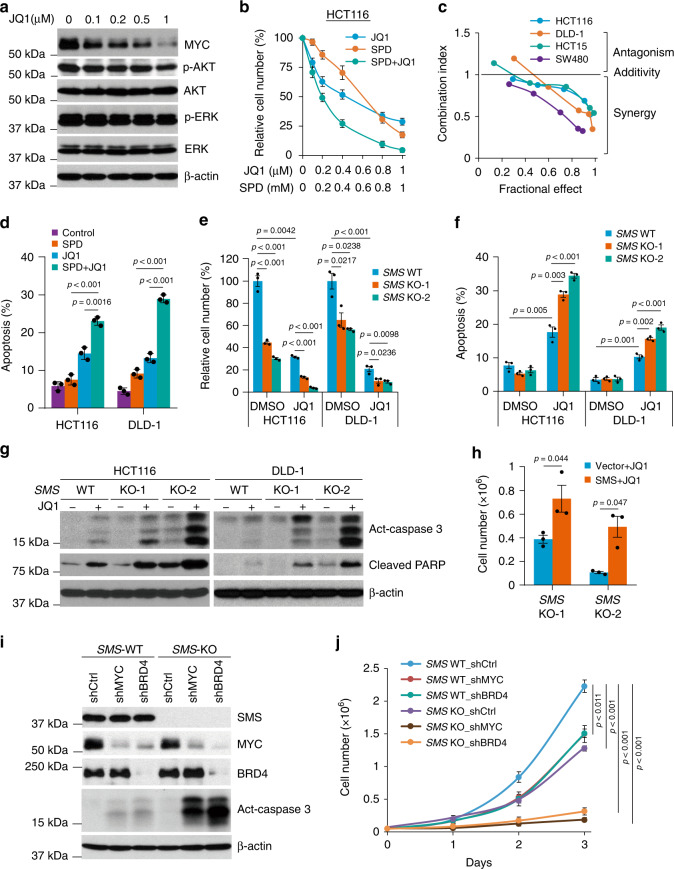
Downregulation of both SMS and MYC synergistically induces apoptosis in CRC cells. **a** HCT116 cells were treated with the indicated concentration of JQ1 for 24 h, followed by western blot analysis for the indicated proteins. **b** HCT116 cells were treated with JQ1 and spermidine (SPD), alone or in combination, with the indicated concentrations for 72 h. Results are expressed as a percentage of viable cell number relative to the value obtained in DMSO-treated control cells. **c** The indicated cells were treated with a combination of JQ1 and SPD. Combination index values were determined using the Chou–Talalay method for drug combinations with a fractional effect between 0.20 and 0.99 (20–99% of cell growth inhibition relative to control). **d** Apoptosis was assessed in HCT116 and DLD-1 cells treated for 72 h with 1 µM JQ1 and 1 mM SPD, alone or in combination, or with DMSO as control. **e** *SMS*-WT or *SMS*-KO HCT116 and DLD-1 cells were assessed for cell growth after 72 h of treatment with 1 µM JQ1 or with DMSO as control. Results are expressed as a percentage of cell number relative to the values obtained with DMSO-treated *SMS*-WT cells. **f** Apoptosis was assessed in *SMS*-WT or *SMS*-KO HCT116 and DLD-1 cells treated with 1 µM JQ1 or with DMSO as control for 72 h. **g** *SMS*-WT or *SMS*-KO HCT116 and DLD-1 cells were treated with 1 µM JQ1 or DMSO for 24 h, followed by western blot analysis for the indicated proteins. **h** *SMS*-KO HCT116 cells with re-expression of SMS or vector control were treated with 1 µM JQ1 for 3 days, followed by counting the number of viable cells. (**i, j**) *SMS*-WT or *SMS*-KO HCT116 cells with stable expression of MYC shRNA (shMYC), BRD4 shRNA (shBRD4) or control shRNA (shCtrl) were analyzed by western blot for the indicated proteins (**i**), or assessed for cell growth (**j**). Data are presented as mean values ±SEM (*n* = 3 independent experiments) in **b**, **d**–**f**, **h** and **j**. The indicated *p*-values were determined by two-tailed unpaired *t* test. Source data are provided as a Source Data file.

Polyamines play an important role in the regulation of cell-cycle progression or apoptosis depending on other environmental signals^10,19,20^. We first investigated the effect of *SMS* deletion on cell-cycle progression and survival. Flow cytometry analysis revealed no significant difference in the distribution of cell-cycle phases and the induction of apoptosis between multiple *SMS*-WT and *SMS*-KO individual clones generated from both HCT116 and DLD-1 cells (Supplementary Fig. 5a–c). Similar results were observed in the mixed stable shRNA-mediated SMS knockdown HCT116 cell populations compared with their control cell populations (Supplementary Fig. 5d, e). Furthermore, addition of exogenous spermidine to the *SMS*-WT HCT116 cells to mimic spermidine accumulation seen in *SMS*-KO HCT116 cells, had also no effect on cell-cycle progression and survival (Supplementary Figs. 4f and 5f, g). These data suggest that the effect of *SMS* deletion with spermidine accumulation on CRC cell growth is regulated by other biological processes. MYC is activated in nearly all CRC^11^ and involved in the regulation of polyamine biosynthesis^1,2^. Similar to *SMS* expression, *MYC* was also overexpressed in all stages of CRC (Supplementary Fig. 6a). Interestingly, *MYC* expression showed a significant positive correlation with *SMS* expression in CRC (Supplementary Fig. 6b), which suggests a potential collaborative link between MYC and SMS in maintaining or promoting CRC tumorigenesis. The small-molecule JQ1 is known to inhibit MYC expression and its transcriptional activity through targeted inhibition of the bromodomain protein BRD4, a key transcriptional regulator of MYC expression^26,27^. In HCT116 cells, JQ1 showed concentration-dependent inhibition of MYC expression, but had no effect on the activated phosphorylation of AKT and ERK kinases (Fig. 3a). Downregulation of MYC expression by JQ1 inhibited CRC cell growth, and this inhibitory effect was synergistic when combined treatment with spermidine in multiple CRC cell lines (Fig. 3b, c). The synergistic growth inhibition was associated with marked induction of apoptosis (Fig. 3d). Similar to spermidine treatment, *SMS* deletion in combination with JQ1 also markedly inhibited cell growth and induced apoptosis with enhanced activation of caspase 3 and induction of cleaved PARP in both HCT116 and DLD-1 cells (Fig. 3e–g), whereas restoration of SMS expression significantly attenuated the growth inhibition in the *SMS*-KO HCT116 cells treated with JQ1 (Fig. 3h). Consistent with the mechanism of action for JQ1, silencing *BRD4* that repressed MYC expression or direct knockdown of MYC expression by shRNAs also dramatically activated caspase 3 and suppressed cell growth in *SMS*-KO HCT116 cells (Fig. 3i, j). Notably, SMS expression was not affected by knockdown of either MYC or BRD4 in *SMS*-WT cells (Fig. 3i). These findings indicate that SMS is not a transcriptional target of *MYC* oncogene, but cooperate with MYC to drive CRC cell growth and survival.

### Co-repression of SMS and MYC markedly induces Bim expression

**Fig. 4:**
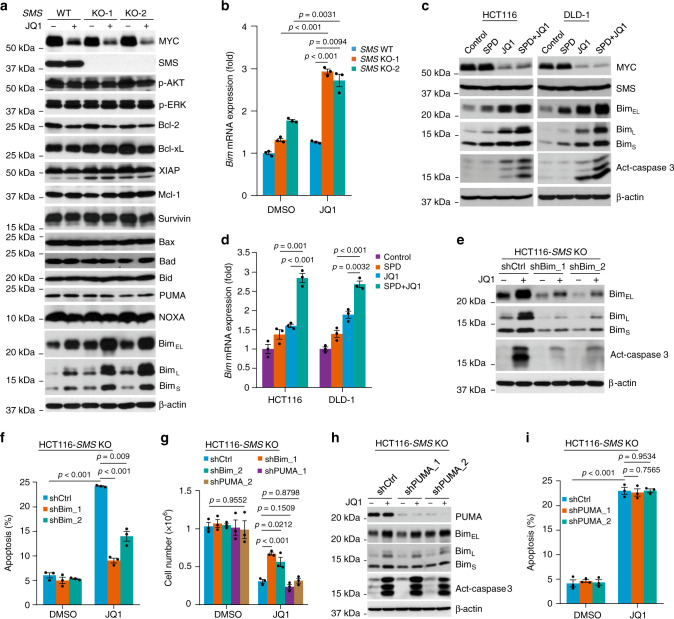
Bim mediates apoptosis induced by co-repressing SMS and MYC expression. **a**, **b** *SMS-*WT or *SMS-*KO HCT116 cells were treated with 1 µM JQ1 or DMSO for 24 h, followed by western blot analysis (**a**) for the indicated proteins or by quantitative RT-PCR analysis (**b**) for *Bim* mRNA expression. **c**, **d** HCT116 and DLD-1 cells were treated with 1 µM JQ1 and 1 mM spermidine (SPD), alone or in combination, or with DMSO as control for 24 h, followed by western blot analysis (**c**) for the indicated proteins or by quantitative RT-PCR analysis (**d**) for *Bim* mRNA expression. **e** *SMS*-KO HCT116 cells with stable expression of two different sets of Bim shRNAs (shBim_1 and shBim_2) or control shRNA (shCtrl) were treated with 1 µM JQ1 or DMSO for 24 h, followed by western blot analysis for the indicated proteins. **f** *SMS-*KO HCT116 cells with stable expression of Bim shRNAs or shCtrl were treated with 1 µM JQ1 or DMSO for 72 h, followed by analysis of apoptosis. **g** *SMS-*KO HCT116 cells with stable expression of Bim shRNAs, PUMA shRNAs, or shCtrl were treated with 1 µM JQ1 or DMSO for 3 days, followed by counting the number of viable cells. **h**, **i** *SMS*-KO HCT116 cells with stable expression of two different sets of PUMA shRNAs (shPUMA_1 and shPUMA_2) or shCtrl were treated with 1 µM JQ1 or DMSO for 24 h followed by western blot analysis for the indicated proteins (**h**), or for 72 h followed by analysis of apoptosis (**i**). All graphic data are presented as mean values ±SEM (*n* = 3 independent experiments). The indicated *p*-values were determined by two-tailed unpaired *t* test in **b**, **d**, **f**, **g** and **i**, or one-way ANOVA test in **g**. Source data are provided as a Source Data file.

To determine the molecular mechanism by which *SMS* deletion in combination with MYC repression by JQ1 synergistically induces apoptosis, we investigated a number of proteins known to be involved in the regulation of apoptosis. Strikingly, we found that the proapoptotic protein Bim^28^, including all three isoforms (Bim_EL_, Bim_L_, and Bim_S_), were markedly upregulated by treatment with JQ1 in multiple *SMS*-KO HCT116 and DLD-1 cell clones, whereas either depletion of SMS in these clones or JQ1 treatment in multiple *SMS*-WT cell clones resulted in moderate induction of the three Bim isoforms (Fig. 4a; Supplementary Fig. 7a). Moreover, the marked induction of Bim expression correlated with maximal activation of caspase 3 and induction of cleaved PARP in the *SMS*-KO clones treated with JQ1 (Fig. 3g; Supplementary Fig. 7a). Similar results were observed in both HCT116 and patient-derived Pt130 primary CRC^29^ cells with stable knockdown of SMS expression as well as in *SMS*-defective SRS lymphoblastoid and fibroblasts (Supplementary Fig. 7b–d). In contrast, expression of other proapoptotic proteins (Bax, Bad, Bid, PUMA, and NOXA) and anti-apoptotic proteins (XIAP, Mcl-1, and survivin) as well as the activated phosphorylation of AKT and ERK kinases were unaffected by JQ1 treatment alone or in combination with *SMS* deletion in HCT116 cells (Fig. 4a). Although some anti-apoptotic proteins such as Bcl-2 and Bcl-xL were slightly downregulated by JQ1, this downregulation was not further enhanced by *SMS* deletion (Fig. 4a). Given that ODC is a transcriptional target of *MYC*, JQ1 treatment significantly inhibited ODC activity, resulting in a decrease in putrescine levels, but had no effect on the levels of spermidine and spermine in HCT116 cells (Supplementary Fig. 8a, b). In addition, *SMS* KO-induced spermidine accumulation was also not affected by JQ1 treatment (Supplementary Fig. 8b). Notably, the ODC inhibitor DFMO, effectively inhibited ODC activity so as to decrease both putrescine and spermidine levels, and lead to a significant accumulation in G1 phase in HCT116 cells (Supplementary Fig. 8a, c, d). However, unlike JQ1, DFMO inhibited Bim expression and had no effect on apoptosis induction in HCT116 cells (Supplementary Fig. 8e, f). Moreover, DFMO did not further enhance JQ1-induced G1 arrest and apoptosis, and showed no synergistic growth inhibition in combination with JQ1 in HCT116 cells (Supplementary Fig. 8d, f–h). Thus, these data indicate that depletion of spermidine by DFMO and accumulation of spermidine by *SMS* deletion respond differentially to MYC repression by JQ1 on CRC cell growth and survival.

The marked induction of Bim protein expression by combination of JQ1 treatment and *SMS* deletion was associated with a profound increase in the level of *Bim* mRNA expression as determined by quantitative real-time (RT) PCR, but *SMS* KO or JQ1 treatment alone only slightly induced expression of *Bim* mRNA (Fig. 4b). These results suggest that SMS and MYC may cooperate to regulate *Bim* transcriptional activity. Similar results were observed by the combination treatment with spermidine and JQ1 in both HCT116 and DLD-1 cells (Fig. 4c, d). Importantly, silencing *Bim* dramatically prevented caspase 3 activation and induction of apoptosis and reversed growth inhibition by JQ1 treatment in *SMS*-KO HCT116 cells (Fig. 4e–g). These effects, however, were not observed by knockdown of PUMA expression (Fig. 4g–i). Together, these findings reveal that the marked induction of apoptosis by *SMS* deletion and JQ1 treatment in CRC cells is largely mediated by Bim.

### *SMS* deletion inhibits FOXO3a acetylation for Bim induction

**Fig. 5:**
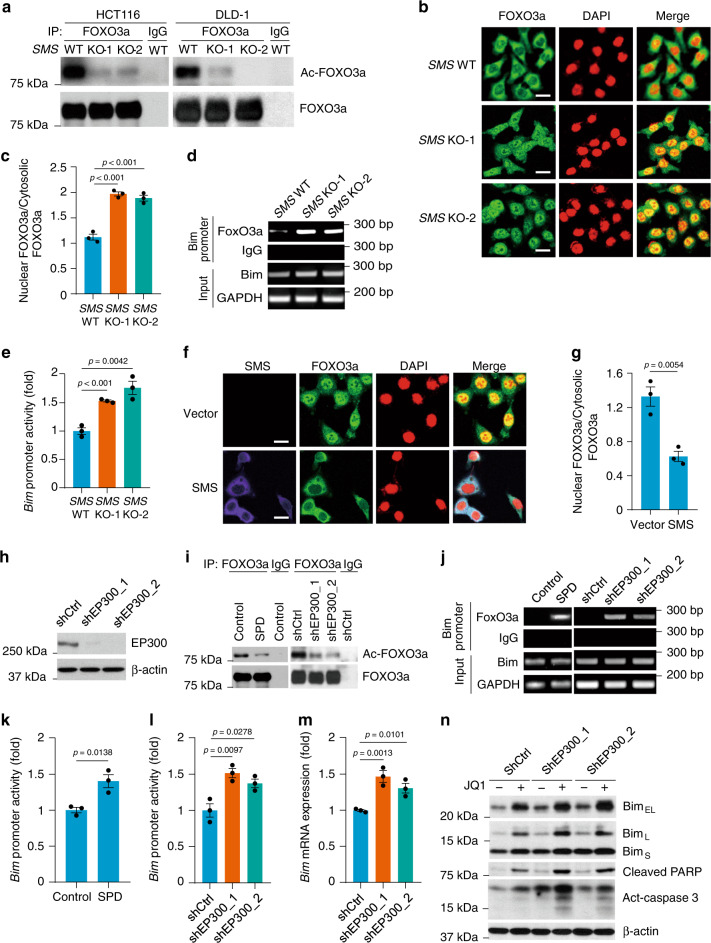
*SMS* deletion induces Bim expression via inhibition of FOXO3a acetylation. **a** *SMS*-WT or *SMS*-KO HCT116 and DLD-1 cell lysates were immunoprecipitated with FOXO3a antibody or IgG as control followed by western blot analysis for the indicated proteins. **b** Representative confocal images of *SMS*-WT or *SMS*-KO HCT116 cells stained for FOXO3a (green) and DAPI (red). **c** The distribution of FOXO3a in the cytosol and nucleus shown in **b** was analyzed using Image J. **d** ChIP analysis of *SMS*-WT or *SMS*-KO HCT116 cell extracts using a FOXO3a antibody or an irrelevant IgG. **e** *Bim* promoter activity was assessed in *SMS*-WT or *SMS*-KO HCT116 cells. **f**, **g** *SMS*-KO HCT116 cells with re-expression of SMS or vector control were assessed by confocal microscopy (**f**) for cellular staining of SMS (purple), FOXO3a (green), and DAPI (red), followed by quantification of FOXO3a distribution in the cytosol and nucleus (**g**). **h** HCT116 cells with stable expression of two different sets of EP300 shRNAs or control shRNA were analyzed by western blot analysis for the indicated proteins. **i**, **j** Cell lysates from HCT116 cells treated with 1 mM spermidine (SPD) or PBS for 24 h, or expressing EP300 shRNAs or control shRNA, were immunoprecipitated with FOXO3a antibody or IgG as control, followed by western blot analysis for the indicated proteins (**i**), or by ChIP analysis (**j**) of FOXO3a interaction with Bim promoter. **k**, **l** Bim promoter activity was analyzed in HCT116 cells treated with 1 mM SPD or PBS as control for 24 h (**k**), or in HCT116 cells expressing EP300 shRNAs or control shRNA (**l**). **m** The expression levels of *Bim* mRNA in HCT116 cells expressing EP300 shRNAs or control shRNA were quantified by RT-PCR analysis. **n** HCT116 cells expressing EP300 shRNAs or control shRNA were treated with 1 µM JQ1 or DMSO for 24 h, followed by western blot analysis for the indicated proteins. Scale bar, 25 µm. All graphic data are presented as mean values ±SEM (*n* = 3 independent experiments). The indicated *p*-values were determined by two-tailed unpaired *t* test. Source data are provided as a Source Data file.

*Bim* is a well-characterized target gene of the transcription factor FOXO3a^30,31^. The transcriptional function of FOXO3a is often regulated by multiple post-translational modifications such as phosphorylation and acetylation^32^. Both AKT-mediated phosphorylation and EP300 acetyltransferase-mediated acetylation of FOXO3a can decrease its DNA-binding efficiency and subsequently lead to its translocation from the nucleus to the cytoplasm^33–37^. A recent study identifies spermidine as a direct EP300 inhibitor^38^. Therefore, we sought to determine whether Bim alteration by *SMS* deletion is regulated by the post-translationally-modified FOXO3a. We found that *SMS* KO did not affect phosphorylation of AKT and its substrate FOXO3a in both HCT116 and DLD-1 cells (Supplementary Fig. 9a). However, the acetylation of FOXO3a was dramatically inhibited in these *SMS*-KO cells, which resulted in FOXO3a transport to the nucleus and binding to *Bim* promoter to increase its transcriptional activity (Fig. 5a–e). Conversely, re-expression of SMS in *SMS*-KO HCT116 cells prevented the inhibition of FOXO3a acetylation and resulted in FOXO3a nucleocytoplasmic transport and repression of Bim promoter activity and protein expression (Fig. 5f, g; Supplementary Fig. 9b–d). In contrast, addition of putrescine or spermine in *SMS*-KO HCT116 cells did not reverse the inhibitory effect on FOXO3a acetylation by *SMS* KO (Supplementary Fig. 9b). However, exposure of HCT116 cells to spermidine or knockdown of EP300 expression inhibited FOXO3a acetylation and increased FOXO3a translocation to the nucleus and binding to *Bim* promoter to activate *Bim* transcription (Fig. 5h–m; Supplementary Fig. 10). Similar to *SMS* deletion or treatment with spermidine (Fig. 4a, c), silencing *EP300* also increased Bim expression and caspase 3 activation, and these effects were further enhanced by treatment with JQ1 (Fig. 5n). Collectively, these data demonstrate that *SMS* deletion-induced elevation of spermidine results in inhibition of EP300-mediated FOXO3a acetylation, which in turn leads to FOXO3a transport to the nucleus, and increases its binding to the *Bim* promoter to stimulate Bim expression.

### MYC-driven miR-19a and miR-19b co-repress Bim expression

**Fig. 6:**
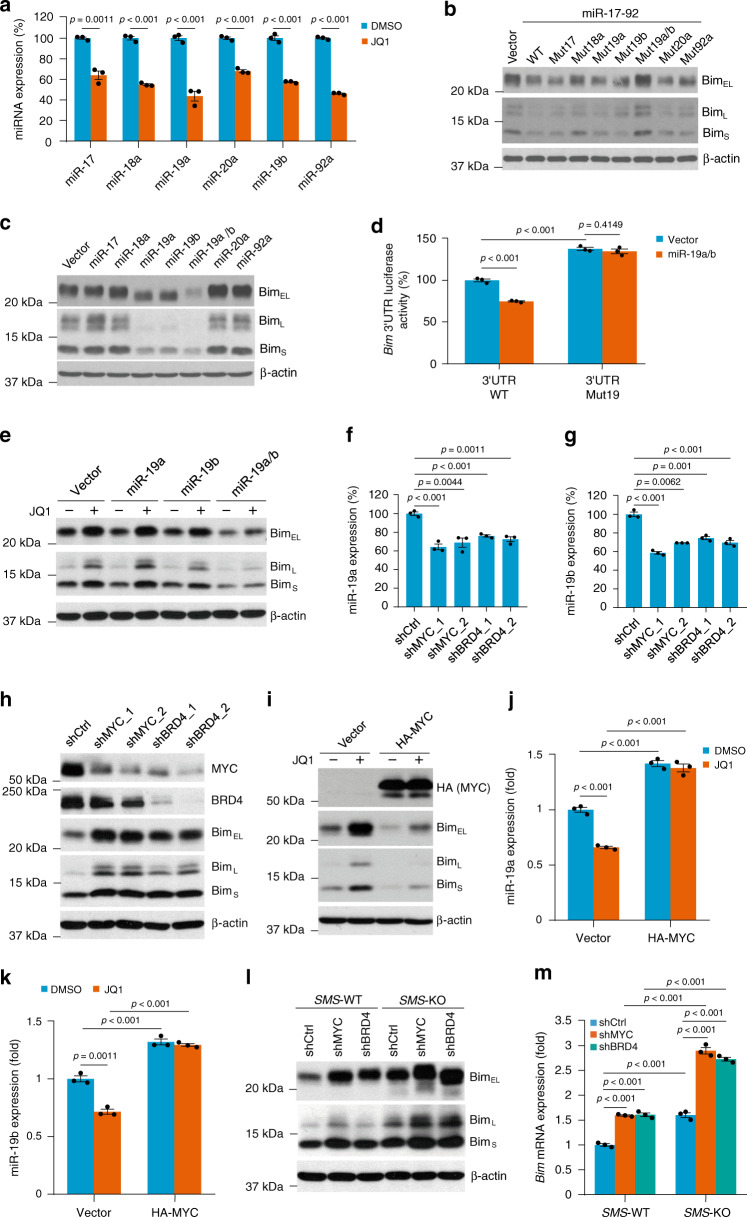
MYC downregulates Bim expression by upregulation of miR-19a and miR-19b. **a** The expression levels of the indicated miRNAs were quantified by RT-PCR analysis in HCT116 cells treated with 1 µM JQ1 or DMSO for 24 h. **b**, **c** Western blot analysis of HCT116 cells with stable expression of miR-17-92 and its mutants (**b**) or the indicated miRNAs (**c**) and their vector control. **d** HCT116 cells with stable expression of miR-19a/b or vector control were co-transfected with a wild-type (WT) or mutant 19 (Mut19) human *Bim* 3′UTR reporter and an internal control *Renilla*-Luc. After 36 h, luciferase activities were measured and normalized. The results are expressed as a percentage of the luc activity found in WT-transfected vector control cells. **e** HCT116 cells with stable expression of miR-19a, miR-19b, miR-19a/b, or vector control were treated with 1 µM JQ1 or DMSO for 24 h, followed by western blot analysis for the indicated proteins. **f**–**h** HCT116 cells with stable expression of two different sets of MYC shRNAs or BRD4 shRNAs, or control shRNA, were analyzed by quantitative RT-PCR for expression of miR-19a (**f**) and miR-19b (**g**), or by western blot (**h**) for the indicated proteins. **i**–**k** HCT116 cells with stable expression of HA-MYC and vector control were treated with 1 µM JQ1 or DMSO for 24 h, followed by western blot analysis (**i**) for the indicated proteins, or by quantitative RT-PCR analysis for expression of miR-19a (**j**) and miR-19b (**k**). **l**, **m** *SMS*-WT or *SMS*-KO HCT116 cells with stable expression of MYC shRNA, BRD4 shRNA, or control shRNA were analyzed by western blot for the indicated proteins (**l**), or by quantitative RT-PCR analysis for *Bim* mRNA expression (**m**). All graphic data are presented as mean values ±SEM (*n* = 3 independent experiments). The indicated *p*-values were determined by two-tailed unpaired *t* test. Source data are provided as a Source Data file.

Although *SMS* deletion induced Bim expression via inhibition of FOXO3a acetylation, the MYC function inhibitor JQ1 could further enhance Bim induction at both the protein and mRNA levels (Fig. 4a, b). These data suggest that MYC may also negatively regulate Bim expression through a mechanism distinct from SMS. MYC has been shown to transcriptionally induce expression of the miR-17-92 cluster, which is a single polycistronic primary transcript that produces six mature microRNAs (miRNAs): miR-17, miR-18a, miR-19a, miR-20a, miR-19b, and miR-92a^39^ (Supplementary Fig. 11a). Several studies identified *Bim* as a key miR-17-92 target gene^40,41^. We first used quantitative RT-PCR to determine the effect of JQ1 treatment on the levels of individual miR-17-92 components in CRC cells. As shown in Fig. 6a, JQ1 effectively repressed expression of all individual miR-17-92 components in HCT116 cells. As expected, delivery of the miR-17-92 cluster that expressed all of the individual miRNAs showed suppression of Bim expression in HCT116 cells (Fig. 6b; Supplementary Fig. 11b). To determine which individual members of miR-17-92 are specifically involved in the regulation of Bim expression, we engineered a 12-nucleotide seed mutation for individual miRNAs within miR-17-92 to abolish the individual miRNA function with minimal disruption to the overall gene structure^42^ (Supplementary Fig. 11a and Table 1). The resulting miR-17-92 mutant with loss of both miR-19a and miR-19b functions was not able to inhibit Bim expression as compared with wild-type miR-17-92 and its other mutants (Fig. 6b). Furthermore, ectopic expression of both miR-19a and miR-19b was required to maximally suppress Bim expression, whereas expression of individual miR-17-92 members had either no or a modest effect on Bim inhibition in HCT116 cells (Fig. 6c; Supplementary Fig. 11c, d). Reporter assays confirmed direct *Bim* 3′ untranslated region (UTR) inhibition mediated by the predicted miR-19 binding site^43^ (Fig. 6d). These data revealed that miR-19a and miR-19b are two major components of miR-17-92 cluster in suppression of Bim expression in HCT116 cells. Notably, expression of either miR-19a or miR-19b showed a modest inhibition of Bim expression, and did not attenuate JQ1-induced Bim expression (Fig. 6e). However, expression of both miR-19a and miR-19b markedly repressed Bim expression and almost completely prevented Bim upregulation by JQ1 (Fig. 6e), indicating that both miR-19a and miR-19b are required to mediate the effect of JQ1 on Bim expression. Similar to the effect of JQ1, silencing *MYC* or *BRD4* in HCT116 cells resulted in a significant decrease in the expression of both miR-19a and miR-19b accompanied by an increase in Bim expression (Fig. 6f–h). Conversely, ectopic expression of MYC significantly increased the expression of both miR-19a and miR-19b with a concomitant decrease in Bim expression, and profoundly abrogated JQ1-induced miR-19a/b downregulation and Bim expression (Fig. 6i–k). Moreover, Bim induction by silencing *MYC* or *BRD4* was further enhanced by *SMS* deletion in HCT116 cells at both the protein and mRNA levels (Fig. 6l, m). Taken together, these findings highlight MYC as a key target of JQ1 responsible for its effect on Bim induction via regulation of miR-19a/b expression. Furthermore, these data also indicate that MYC and SMS cooperate to regulate Bim expression in CRC cells through distinct pathways.

### Co-repressing SMS and MYC expression causes tumor regression

**Fig. 7:**
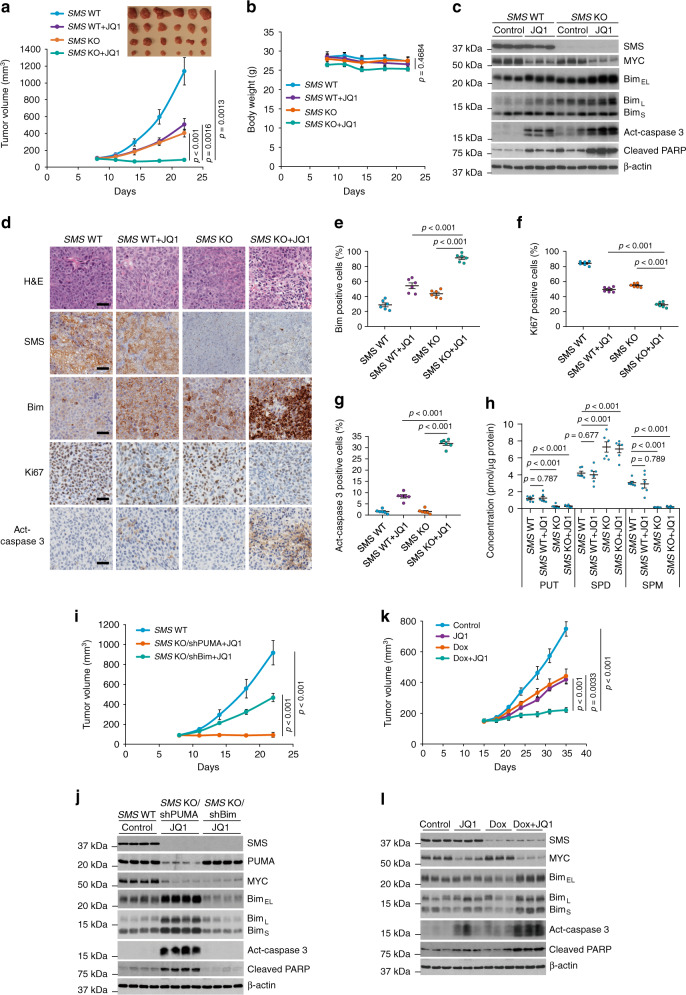
Silencing *SMS* in combination with JQ1 markedly suppress CRC tumor growth. **a**, **b** Mice bearing *SMS*-WT or *SMS*-KO HCT116 xenograft tumors were treated daily with vehicle or JQ1 at 50 mgkg^−1^. The tumor volume (**a**) and body weight (**b**) are presented as mean values ±SEM (*n* = 6 mice/group). **c**, **d** Representative tumors from mice euthanized 6 h after the final treatment with JQ1 or vehicle control as in **a** were analyzed by western blot (**c**) for the indicated proteins, or by H&E staining and IHC staining (**d**) for the indicated proteins. Scale bar, 30 µm. **e**–**g** Bim (**e**), Ki67 (**f**), and Act-caspase 3 (**g**) positive cells shown in **d** were quantified using Image J. The results are presented as the mean percentage of the positive cells ±SEM (*n* = 6 mice/group). **h** The levels of putrescine (PUT), spermidine (SPD), and spermine (SPM) in the tumors from mice (*n* = 6 mice/group) euthanized 6 h after the final treatment with JQ1 or vehicle control as in **a** were determined by LC-MS. **i** Mice bearing *SMS*-WT or *SMS*-KO HCT116 xenograft tumors with stable expression of Bim shRNA or PUMA shRNA were treated daily with vehicle or JQ1 at 50 mgkg^−1^. The tumor volume was presented as mean values ±SEM (*n* = 6 mice/group). **j** Representative tumors from mice euthanized 6 h after the final treatment with JQ1 or vehicle control as in **i** were analyzed by western blot. **k** Mice bearing Pt130 xenograft tumors expressing tet-inducible SMS shRNA were maintained with or without doxycycline (Dox, 0.5 mgml^−1^) in the drinking water and treated daily with vehicle or JQ1 at 50 mgkg^−1^. Dox was given to mice until the JQ1 treatment was completed. The tumor volume was presented as mean values ±SEM (*n* = 6 mice/group). **l** Representative tumors from mice euthanized 6 h after the final treatment with JQ1 and Dox, alone and in combination, as in **k** were analyzed by western blot. The indicated *p*-values were determined by two-tailed unpaired *t* test in **a**, **e**–**i**, and **k**, or one-way ANOVA test in **b**. Source data are provided as a Source Data file.

**Fig. 8:**
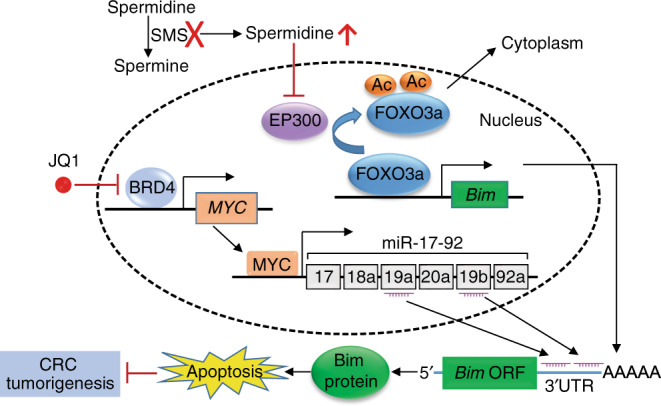
A model for SMS and MYC cooperation in CRC tumorigenesis. Co-repressing Bim expression by the SMS/spermidine/EP300/FOXO3a and MYC/miR-19a/b signaling pathways is required for maintenances of CRC cell survival and tumor growth. Combined inhibition of both pathways synergistically induces Bim expression and apoptosis, and causes tumor regression.

The marked induction of apoptosis associated with CRC cell growth inhibition by *SMS* deletion in combination with MYC repression by JQ1 in vitro suggests that targeting both SMS and MYC signaling may be an effective strategy for CRC therapy. To explore this possibility, nude mice bearing established *SMS-*WT or *SMS-*KO HCT116 xenograft tumors were treated with JQ1 or vehicle control. Previous studies showed that treatment with JQ1 at 50 mg/kg could effectively downregulate MYC expression in multiple xenograft tumor models^44,45^. Administration of JQ1 with 50 mg/kg daily for 2 weeks slowed tumor growth in mice with *SMS-*WT HCT116 xenografts without significant weight loss (Fig. 7a, b). *SMS* deletion also showed a modest antitumor effect (Fig. 7a). Neither *SMS* deletion nor JQ1 completed prevented tumor growth. However, JQ1 in combination with *SMS* deletion synergistically suppressed the growth of the tumor xenografts and caused tumor regression (Fig. 7a). Western blot and IHC analyses showed that JQ1 effectively inhibited MYC expression and induced a modest increase in Bim expression with concomitant inhibition of cell proliferation as measured by Ki67 and apoptosis induction as measured by cleaved (activated) caspase 3 and PARP (Fig. 7c–g). Similar to *SMS* KO-induced spermidine accumulation as seen in vitro, *SMS* deletion also produced the excessive level of spermidine in the xenograft tumors (Fig. 7h), which was associated with a modest Bim induction and inhibition of cell proliferation (Fig. 7c–f). However, *SMS* deletion in combination with JQ1 treatment synergistically induced Bim expression and apoptosis associated with tumor regression (Fig. 7a, c–g). Notably, silencing *Bim*, not *PUMA*, largely prevented the tumor growth inhibition and caspase 3 activation by combination of *SMS* deletion and JQ1 treatment (Fig. 7i, j). Similar observations with profound antitumor effect and caspase 3 activation induced by silencing *SMS* in combination with JQ1 treatment were found in the Pt130 primary CRC xenograft model in which mice were treated with doxycycline in drinking water for induction of *SMS*-targeting shRNAs (Fig. 7k, l). Thus, these results confirm the relevance of the tissue culture data to in vivo models, and implicate Bim as a key target that mediates the synergistic antitumor effect by combined inhibition of SMS and MYC signaling pathways in CRC (Fig. 8).

## Discussion

Polyamine biosynthesis is frequently dysregulated in human cancers, and in particular, is associated with early-stage CRC development^12^. Recent studies have brought polyamine metabolism back to the limelight, providing a strong link between polyamine metabolic pathway and oncogenic signaling pathways in tumorigenesis^1,2^. In this study, we uncover an important function of SMS in cooperation with MYC to repress Bim expression through distinct regulatory pathways, and highlight the downregulation of Bim as a key survival signal downstream of oncogenic MYC and SMS signaling to facilitate CRC tumor growth (Fig. 8).

Our study provides several insights into the biology and therapeutic relevance of cooperative SMS and MYC signaling in CRC tumorigenesis. First, our study suggests that overexpression of SMS plays an important role in balancing cellular spermidine levels that are a necessary adaptation for CRC tumorigenesis. Polyamine homeostasis is tightly regulated in normal cells and required for cell growth and tissue regeneration. Decreases in tissue spermidine concentrations resulting from a decline in polyamine biosynthesis occur with age in model organisms as well as in humans^46–49^. In contrast, upregulation of the biosynthetic activities of polyamine-producing enzymes such as ODC, SRM, SAMDC, and SMS by oncogenic signaling results in increased levels of polyamines, which are thought to play important roles in the maintenance of the transformed phenotype and tumor development^2,3,18^. However, excessive polyamine accumulation can itself have major deleterious effects on cell viability^19,20,50^. For instance, ODC-overproducing L1210 leukemic cells produced a massive intracellular level of spermidine that is cytotoxic independent of its oxidative metabolites^51^. Conversely, reduction of spermidine levels by treatment with the ODC inhibitor DFMO, attenuates apoptosis induced by different cell death stimuli^19,52^. In addition, several studies have demonstrated that spermidine production resulting from SRM expression or direct treatment with spermidine significantly suppress cell growth in vitro and in vivo models of CRC^53^ and hepatocellular carcinoma^54^, which is likely mediated by induction of autophagy through transcriptional and epigenetic regulation^48,55^. Similar to these findings, we found that SMS is overexpressed in CRC throughout tumorigenesis, and target disruption of *SMS* caused over-accumulation of spermidine that correlates with CRC cell growth inhibition. This correlation is supported by our findings that: (1) increased intracellular spermidine could inhibit CRC cell growth in a concentration-dependent manner; and (2) restoration of spermine or putrescine levels in SMS-deficient CRC cells did not alter spermidine accumulation or reverse cell growth inhibition by *SMS* deletion, but re-expression of SMS resulting in a marked decrease in the spermidine level did restore cell growth. In contrast to the detrimental consequence of elevated spermidine in *SMS-*KO CRC cells, normal cells such as embryonic stem cells and fibroblasts or skin fibroblasts lacking SMS grow at a normal rate, although the spermidine levels are also increased in the SMS-deficient normal cells^24,25,56^. These data suggest that normal cells, with properly regulated polyamine homeostasis, can tolerate a certain level of spermidine which is beneficial for their proliferation. In CRC cells, however, the spermidine level is much higher than in normal cells due to the upregulation of polyamine biosynthetic enzymes from oncogenic signaling^2,3,12^. As such, overexpression of SMS, converting spermidine to spermine, is likely required to prevent the spermidine level from extending beyond the toxic threshold, and still maintain a relatively high level of spermidine favorable to support tumor growth. Thus, genetic ablation of *SMS* completely inhibits SMS function and induces excessive accumulation of spermidine, leading to growth inhibition in CRC. Taken together, our findings highlight an important role of SMS in spermidine homeostasis in CRC and offer mechanistic insights into the cellular consequences of spermidine imbalance in CRC tumorigenesis.

Second, our study reveals a cooperative link between SMS and MYC signaling to maintain CRC cell survival through co-repression of Bim expression. Loss-of-function mutations in *SMS* gene result in SRS, an X-linked intellectual disability syndrome^4,5^. However, the role of SMS in cancer is less characterized and understood. As indicated above, our work suggests that SMS overexpression is required to reduce spermidine level below the cytotoxic threshold and support tumor growth in CRC. Analysis of SMS expression at the mRNA or protein level (Fig. 1b–f) associated with patients’ survival did not identify a significant correlation between high SMS expression and short survival in CRC. In subtype analysis by tumor stage (Supplementary Fig. 1a, b) or cell differentiation status, we also did not detect significant association between SMS expression and CRC prognosis. However, due to the small sample size used in our study, additional large studies, particularly studies with subgroups for therapy regimens and environmental-genetic interactions, are needed to validate our findings. Aberrant expression of MYC is observed in many human cancers including CRC^11^, but the prognostic value of MYC in CRC remains debatable. A recent report using a meta-analysis shows that MYC is not significantly associated with CRC prognosis^57^. We found that MYC expression is significantly and positively associated with SMS expression in CRC, although SMS expression is not regulated by MYC. We divided CRC tissue samples from the TCGA dataset (Supplementary Fig. 6b) in two groups: high SMS/high MYC and low SMS/low MYC expression, and performed a Kaplan–Meier survival analysis between two groups. We found that CRC patients with high expression of both SMS and MYC were not significantly associated with poor survival. Dysregulation of MYC is a consequence of mutations in *APC*, a central hub in early colorectal carcinogenesis^58^. In a mouse model of Min-Apc intestinal tumorigenesis, mice that overexpressed SMS display similar colon tumor multiplicity and size compared with multiple intestinal neoplasia as seen in the Min mice^59^. Together, these data suggest that SMS alone does not promote CRC tumorigenesis. Nevertheless, our data demonstrated that SMS cooperates with MYC to maintain CRC cell survival. Genetic and/or pharmacologic inhibition of either SMS or MYC activity moderately suppressed CRC cell growth associated with no or moderate induction of apoptosis. However, combined inhibition of SMS and MYC signaling synergistically repressed cell growth and induced apoptosis. This synergy results from inhibition of the two parallel pathways that cooperate to downregulate their common downstream target, the proapoptotic protein Bim, through distinct regulatory mechanisms. We showed that SMS blocks Bim transcription by reducing spermidine-mediated inhibition of FOXO3a acetylation, whereas MYC downregulates Bim expression post-transcriptionally by inducing both miR-19a and miR-19b. Inhibition of either SMS or MYC signaling pathway is insufficient to induce Bim expression, but combined inhibition of both pathways is required for maximal induction of Bim expression and apoptosis in CRC cells. Importantly, silencing *Bim* expression prevents apoptosis and tumor growth in response to inhibition of both pathways. Thus, the Bim protein integrates the survival signaling by SMS/spermidne/FOXO3a and MYC/miR-19a/b pathways in CRC cells. Our findings indicate that inhibition of Bim function by activation of SMS and MYC signaling pathways plays a crucial role in maintaining the transformed phenotype of CRC.

Third, our study suggests that concurrent inhibition of SMS and MYC signaling is a promising therapeutic strategy for CRC. The Cancer Genome Atlas data indicate that MYC-dependent transcription is activated in nearly all CRC^11^, and thus, MYC is an attractive therapeutic target for CRC. Although there is no clear ligand-binding domain for targeting MYC, emerging evidence demonstrate that small-molecule compounds such as JQ1, which targets the bromodomain and extra-terminal domain (BET) family epigenetic readers, act as alternative approaches to inhibit MYC activity^26,27^. Nevertheless, the efficacy of BET inhibitors in CRC is modest in general, suggesting that CRC tumors are intrinsically resistance to BET inhibition. A recent study showed that activation of the RAS/RAF/MAPK pathway is a potential mechanism that mediates intrinsic resistance to JQ1 in CRC cell cultures and xenograft models^60^. Here, we identify the dysregulated polyamine biosynthetic enzyme SMS as a key factor to alter the therapeutic response to JQ1 in CRC. Our data showed that genetic deletion of *SMS* causes a reprograming of polyamine metabolism with over-accumulation of spermidine, which sensitizes CRC cells to JQ1 as demonstrated by the synergistic induction of apoptosis and tumor regression. We revealed that the synergistic antitumor effect by combined inhibition of SMS and MYC signaling pathways is attributed to their mechanistic convergences on the regulation of Bim expression, underscoring the importance of apoptosis regulators in mediating the therapeutic response to CRC. Thus, the crosstalk between SMS and MYC signaling appears to be an intrinsic phenotype of CRC. Our findings indicate that SMS expression may serve as a predictive marker for MYC-targeted CRC therapies by using the BET inhibitors. Several SMS inhibitors such as *N*-(3-aminopropyl)cyclohexylamine, 5′-methylthioadenosine and decarboxylated *S*-adenosylhomocysteine have been created, but these inhibitors show little promise as a chemotherapeutic agent owing to the limited potency and selectivity of the compounds, the slow turnover of polyamines and the autoregulation of the polyamine pathway^1,61,62^. Clinically effective SMS inhibitors remain to be developed. Such SMS inhibitors in combination with BET inhibitors may hold great promise in the treatment of CRC affected by aberrations of both SMS and MYC-mediated signaling pathways.

## Methods

### Cell lines, tissue samples, and chemicals

Human HCT116, DLD-1, HCT15, and SW480 cell lines were obtained from the American Type Culture Collection (ATCC). HCT116 cells were cultured in McCoy’s medium (Sigma). DLD-1 and HCT15 cells were cultured in RPMI-1640 medium (Sigma). SW480 cells and patient-derived Pt130 primary CRC cells^29^ were cultured in DMEM medium (Sigma). All media were supplemented with 10% FBS (Sigma), streptomycin (100 µgml^−1^) and penicillin (100 unitsml^−1^). In experiments with addition of polyamines to the cultures, 1 mM aminoguanidine was included in the medium to inhibit amine oxidase present in the serum^51^. All cell lines were subjected to regular mycoplasma testing via PCR using e-Myco Plus kit (iNtRON Biotechnology) and underwent short tandem repeat (STR) profiling (Genetica). The SRS lymphoblastoid (3811) and fibroblast (CMS1850B, CMS25081A) cell lines and their control cell lines (CMS25378, CMS25400A) were derived from three SRS patients and two healthy male donors. The SRS lymphoblastoid cells were grown in RPMI-1640 medium supplemented with 15% FBS, streptomycin (100 µgml^−1^) and penicillin (100 unitsml^−1^). The SRS fibroblast cells were grown in DMEM medium supplemented with 10% FBS, 1% sodium pyruvate, 1% non-essential amino acids, streptomycin (100 µgml^-1^) and penicillin (100 unitsml^−1^). The primary colon tumors and adjacent normal control tissues were collected with informed consent from patients (pt2893, pt2405, pt2769, pt2647, pt2751, and pt2293) who had undergone surgery resections at the University of Kentucky Markey Cancer Center. Data regarding the CRC stage and pathology status are listed in Supplementary Fig. 1c. Experiments were performed under a protocol approved by the University of Kentucky Institutional Review Board. JQ1 was purchased from MedChem Express. Putrescine (P5780), spermidine (S2626), spermine (S4264), DFMO (D193), aminoguanidine (#396494) and (2-Hydroxypropyl)-β-cyclodextrin (H107) were obtained from Sigma.

### Plasmids and transfection

The human *SMS* was amplified by PCR using a HCT116 cDNA library, and then subcloned into the pLenti6.3 vector^63^. The human *Bim* promoter (position −763/+29)^31^ was amplified from HCT116 genomic DNAs, and then cloned into the pGL3 basic reporter vector that contains firefly luciferase (Promega). The human *Bim* 3′UTR WT fragment as reported^43^ was amplified from a HCT116 cDNA library, and then inserted into a region immediately downstream of the firefly luciferase gene in pGL3 control reporter vector (Promega). The *Bim* 3′UTR mutant was generated using the QuikChange XLII site-directed mutagenesis kit (Stratagene). The pcDNA3-HA-MYC (#74164), MSCV-mir-17-92 (#64100), MSCV-miR-18a (#64228), MSCV-miR19a/b (#24827), MSCV-miR-19b (#23275), MSCV-miR-20a (#24709), and MSCV-miR-92a (#64092) were purchased from Addgene. The miR-17 or miR-19a fragment was amplified using the MSCV-miR-17-92 or MSCV-miR19a/b as a template respectively, and then cloned into the MSCV vector. The miR-17-92Mut17, miR-17-92Mut18a, miR-17-92Mut19a, miR-17-92Mut19b, miR-17-92Mut19a/b, miR-17-92Mut20a, and miR-17-92Mut92a were generated using the QuikChange XLII site-directed mutagenesis kit. The loss of miR-17, miR-18a, miR-19a, miR-19b, miR-20a or miR-92a expression and the intact expression level of the remaining miR-17-92 components were validated using the TaqMan MicorRNA assays (4427975, Applied Biosystems). All primers used are listed in Supplementary Table 1. All sequences were verified by automated DNA sequencing. For transient expression of SMS, cells were infected with lentivirus for 48 h using the pLenti6.3-SMS plasmid. For stable expression of MYC, cells were transfected with pcDNA3-HA-MYC using Lipofectamine 3000 according to the manufacturer’s protocol (Thermo Fisher Scientific), followed by selection with G418 (500 µgml^−1^) for 10 days.

### Gene silencing by shRNA

The lentiviral shRNAs against human SMS, Bim, PUMA, EP300, MYC, and BRD4 were cloned into pLKO.1 or Tet-pLKO.1 vector (Addgene), and their sequences are listed in Supplementary Table 2. The Non-Target control shRNA (SHC002) was from Sigma. To establish stable transfectants with knockdown of specific protein expression, cells were infected with lentivirus using the indicated shRNA constructs followed by selection with puromycin (2 µgml^−1^, for SMS and EP300 shRNAs) or hygromycin (250 µgml^−1^, for Bim, PUMA, MYC, and BRD4 shRNAs) for 7–10 days^63^.

### Generation of human *SMS* KO cell lines

A short guide RNA, 5′-GATACTGGCCCACCGCCGAC-3′, was designed to target exon 5 of human *SMS* using the CRISPR designing tool^21^ (https://zlab.bio/guide-design-resources). The gRNA was cloned into the Cas9-encoding PX459 vector (#62988, Addgene), and transiently transfected into the cells using Lipofectamine 3000. For *SMS* WT, cells were transfected with non gRNA-Cas9-encoding pX459 vector. Single cells were selected by serial dilution followed by puromycin (2 µgml^−1^) treatment for one week. Single cell colonies were screened for internal deletion or insertion by sequencing the PCR fragments using the following primers: 5′-AGGGAAATGCAGTGTATGAGC-3′ (forward) and 5′-AGAGGGACAAAGTGGACTGG-3′ (reverse).

### Measurement of intracellular polyamines

Polyamines were extracted according to the protocol described by Byun et al.^64^ with some modifications. Cell pellets were reconstituted in 100 µl of 0.04% heptafluorobutyric acid, and 100 µl of d20-spermine (1.6 µM) was added as an internal standard. Protein was precipitated and the supernatant was collected and adjusted to pH 9.0 by adding sodium carbonate buffer. Derivatization of amine was performed by adding 50 µL of isobutyl chloroformate followed by incubating at 35°C for 15 min. After cooling, the solution was extracted with 4 mL of ethyl acetate, and the organic solvent was evaporated under a gentle steam of nitrogen at room temperature. The residue was reconstituted using 100 µl of methanol-water (50:50, v/v) solution, and a 2-µl aliquot was injected into the LC-MS system with an Ultimate 3000 ultra-high performance liquid chromatography and a Q-exactive mass spectrometer (Thermo Fisher Scientific). Separation was performed on a Kinetex C18 reversed-phase column (2.6 mm × 100 mm, 2.1 μm) with gradient elution (A, 0.1% formic acid; B, 0.1% formic acid in acetonitrile). Mass spectrometric detection was performed by electrospray ionization in positive mode with source voltage maintained at 4.3 kV. The concentrations of polyamines were calculated based on the LC-MS peak area ratios of analytes to d20-spermine. Levels of polyamines were normalized to protein amount.

### Quantitative RT-PCR analysis

Total cellular RNA was isolated using the RNeasy plus mini kit (Qiagen). Equal amounts of RNA were used as templates for all reactions. cDNA was generated with the SuperScript III First Strand Synthesis System (Invitrogen). RT-PCR was performed with a StepOne RT-PCR system (Applied Biosystems) using Maxima SYBR Green/ROX qPCR Master Mix (Thermo Fisher Scientific). The PCR primers used are: *Bim*, 5′-CTCGGACTGAGAAACGCAAG-3′ (forward) and 5′-CGCAGGCTGCAATTGTCTAC-3′ (reverse); *GAPDH*, 5′-ACAACTTTGGTATCGTGGAAGG-3′ (forward) and 5′-GCCATCACGCCACAGTTTC-3′ (reverse). *GAPDH* was used as an internal control for normalization, and the relative expression level was calculated by the comparative CT (^ΔΔ^CT) method. For quantification of miRNA expression, the miRNAs were isolated using the mirVana miRNA isolation kit (Thermo Fisher Scientific). TaqMan probes (#4427975) were used according to the manufacture’s protocol (Applied Biosystems) and the CT values were normalized to U6. Each experiment was performed in triplicate and repeated at least three times.

### Luciferase reporter assay

Cells (1 × 10^5^) were co-transfected with 0.3 µg of the *Bim* promoter reporter, *Bim* 3′UTR reporter or *Bim* 3′UTR mutant reporter together with 0.03 µg of pHRL-TK *Renilla* luciferase control vector using Lipofectamine 3000. Thirty-six hours post-transfection, firefly and *Renilla* luciferase activities were measured using a dual-luciferase assay kit (Promega). The firefly luciferase activity for each sample was normalized based on transfection efficiency as determined by *Renilla* luciferase activity. Each experiment was performed in triplicate and repeated at least three times.

### ChIP assay

ChIP analysis was performed according to the protocol described by Nowak et al.^65^ with some modifications. Cells (5 × 10^6^) were cross-linked with 1% formaldehyde for 15 min at room temperature, then lysed in L1 buffer (50 mM Tris-HCl, pH 8.0, 2 mM EDTA, 0.1% IGEPAL, 10% glycerol, 1 mM dithiothreitol, 1 mM phenylmethylsulfonyl fluoride and protease inhibitor cocktail) on ice. Nuclei were pelleted by centrifugation and resuspended in ChIP lysis buffer (50 mM Tris-HCl, pH 8.0, 10 mM EDTA, 1% SDS). Chromatin was subjected to sonication and then immunoprecipitated with FOXO3a antibody (sc-48348, Santa Cruz Biotechnology) or an irrelevant immunoglobulin G (IgG) overnight, followed by incubation with a 50% slurry of protein G sepharose/salmon sperm DNA (Thermo Fisher Scientific) for 3 h at 4 °C. Bound DNA–protein complexes were eluted, and crosslinks were reversed after a series of washes. Purified DNA was resuspended in TE buffer (10 mM Tris-HCl, pH 8.0, 1 mM EDTA) for PCR analysis. The primers used for *Bim* promoter are 5′-TACTCCGGTAAACACGCCAG-3′ (forward) and 5′-CGGAGCGAAGTGAAACCTG-3′ (reverse).

### Immunoprecipitation and western blot analysis

Cells were lysed in NP-40 lysis buffer (50 mM Tris-HCl, pH 7.5, 150 mM NaCl, 1 mM EDTA, 1% NP-40, 10% glycerol, protease, and phosphatase inhibitor cocktail). Protein concentrations were measured using the BCA protein assay reagent (Thermo Fisher Scientific). The cell lysates (500 µg protein) were immunoprecipitated with FOXO3a antibody (#12829, Cell Signaling Technology) overnight followed by incubation with a 50% slurry of protein G sepharose beads for 3 h at 4 °C. The beads were washed three times with the lysis buffer, and the immunoprecipitated protein complexes were resuspended in 2× Laemmli sample buffer followed by western blot analysis. For western blot analysis, equal amounts of protein were resolved by SDS-PAGE, transferred to PVDF membranes, immunoblotted with specific primary and secondary antibodies, and detected using chemiluminescence (GE Healthcare). A list of all antibodies used in this work and dilutions can be found in Supplementary Table 3. Contrast of western blot images was adjusted using Adobe Photoshop and uncropped and unprocessed scans are found in the Source Data file.

### ODC activity assay

ODC activity was measured as release of CO_2_ from L-[1-C^14^] ornithine^52^. Cells were lysed in ODC lysis buffer (25 mM Tris-HCl, pH 7.5, 1 mM EDTA, 1 mM DTT, 0.1% Triton X-100, protease, and phosphatase inhibitor cocktail). Protein concentrations were measured using the BCA protein assay reagent (Thermo Fisher Scientific). Cell lysates (100 µg protein) were incubated at 37 °C for 1 h after addition of 0.5 μCi L-[1-C^14^] ornithine hydrochloride (Moravek) and 100 mM Tris-HCl buffer (pH 7.5) containing 0.4 mM l-ornithine, 0.4 mM pyridoxal phosphate, 1 mM EDTA, and 4 mM DTT. Released CO_2_ was captured with a 3-MM chromatography paper (Thermo Fisher Scientific), which was soaked with 50 µl SOLVABLE (PerkinElmer) and put on the top of a glass tube and closed with rubber cap. Reactions were terminated by addition of 2 M citric acid. The 3-MM paper was transferred to a scintillation vial, and 5 mL scintillation fluid were added. Liquid scintillation radioactivity was measured by scintillation counter (LS6500, Beckman Coulter). ODC activity was determined as picomoles of CO_2_ released (cpm) per milligram of protein per hour.

### Cell viability assay

Cells (5 × 10^4^/well) were seeded in six-well plates in triplicate. After 24 h, cells were treated with the indicated drugs and incubated at 37 °C. The cells were cultured for 3 days and the number of viable cells was counted using the Vi-CELL XR 2.06 (Beckman Coulter). For the combination studies, the synergy was assessed using the combination index (CI) according to the Chou-Talalay method^66^ using CompuSyn software (version 1.0.1). Generally, CI values of <1 are taken to indicate synergistic interaction between drugs, and CI values of > 1 indicate no interaction (drug antagonism). Each experiment was performed in triplicate and repeated at least three times.

### 3D cell culture

Cell suspension (3 × 10^4^/250 μl) in the appropriate medium was seeded on top of a thin layer of Matrigel (BD Biosciences) in 24-well plates, and incubated at 37 °C for 30 min, followed by the addition of 250 μl medium containing 10% Matrigel (BD Biosciences) over the cell suspension. The medium was changed every 2 days. Cell spheres were photographed using a Nikon Eclipse Ti-E inverted microscope. The sphere sizes from 50 randomly chosen spheroids were measured using Nikon NIS-Elements AR software (version 5.00.00).

### Cell cycle and apoptosis analysis

Cells were plated in 100-mm dishes. After 24 h, cells were treated as indicated in the figure legends. Both adherent and floating cells were harvested. For cell-cycle analysis, cell nuclei were prepared and stained with ethidium bromide^63^. Cell cycle phase distribution was determined by flow cytometry. For apoptosis, cells were analyzed by flow cytometry using the FITC Annexin V Apoptosis Detection Kit according to the manufacturer’s protocol (Thermo Fisher Scientific). The flow cytometry gating strategies are shown in Supplementary Fig. 12.

### Immunofluorescence

Cells (5 × 10^4^) grown on coverslips were fixed with 4% paraformaldehyde in PBS for 15 min, permeabilized in 0.2% Triton X-100 and 0.5% BSA in PBS for 5 min and then blocked with 4% BSA in PBS for 10 min. The cells were incubated overnight at 4 °C with the indicated primary antibody. After three washes with 0.05% Triton X-100 in PBS, cells were incubated with the indicated secondary antibody for 1 h. Cells were then washed three times, mounted with DAPI containing mounting medium (H-1200, Vector Laboratories), viewed and photographed with a Nikon A1^+^-Ti2 confocal microscope. For the study of FOXO3a distribution in cells, the intensities of FOXO3a in nucleus and whole cells (n = 30) were quantified separately by Image J (version 1.8.0). The levels of FOXO3a in the cytosol were calculated by subtracting the levels of nuclear FOXO3a from that of whole cells. The Primary and secondary antibodies used in this assay are listed in Supplementary Table 3.

### IHC staining

The CRC tissue microarrays were established by the Biospecimen Procurement and Translational Pathology Shared Resource Facility of the University of Kentucky Markey Cancer Center, and contain 57 pairs of tumor and adjacent normal control tissues collected from patients with CRC who had undergone surgery resections at the Markey Cancer Center. Tissue sections were deparaffinized, rehydrated, and treated with hydrogen peroxide. Antigen retrieval was performed using citrate buffer (pH 6.0) in a steamer. Tissue sections were first blocked with avidin, biotin, and 5% normal goat serum and then incubated in a humidified chamber at 4 °C overnight with antibodies as indicated in Supplementary Table 3. The samples were then incubated with biotin-labeled goat anti-rabbit secondary antibody and subsequently with horseradish peroxidase-avidin complex (Vector Laboratories). Antibody-associated staining was visualized using diaminobenzidine substrate solution and the tissue sections were then counterstained with hematoxylin. The immunoreactivity was scored blindly according to the value of immunoreaction intensity (none = 0; weak = 1; intermediate = 2; and strong = 3) and the percentage of tumor cell stained (none = 0; <10% = 1; 10–50% = 2; >50% = 3) using a semi-quantitative seven-tier system^63^. The intensity and percentage values were added to provide a final immunoreactivity score ranging from 0 to 6.

### Animal studies

Male and female athymic nude mice (5–6 weeks old) were purchased from Taconic, and maintained and treated under AAALAC-accredited specific pathogen-free housing vivarium and care, and veterinary supervision following standard guidelines for temperature and humidity with 12/12 light cycle. Experiments were carried out under a protocol approved by the University of Kentucky Institutional Animal Care and Use Committee. Xenograft tumors were established by subcutaneously injecting CRC cells (2 × 10^6^/mouse for HCT116; 1 × 10^7^/mouse for Pt130) in a 1:1 mixture of media and Matrigel (BD Biosciences). Mice were randomized among control and treated groups (*n* = 6/group) when tumors were well-established (~160 mm^3^). JQ1 was first dissolved in DMSO and diluted to 9 vol of 10% β-cyclodextrin in sterile saline, and administrated through intraperitoneal injection^60^. Mice bearing xenograft tumors were treated daily with vehicle or JQ1 at 50 mgkg^−1^. For tet-inducible SMS shRNA expression, mice received doxycycline (0.5 mgml^−1^) in the drinking water^67^. Tumor dimensions were measured using a caliper. The tumor volumes were calculated as mm^3^ = *π*/6 × larger diameter (smaller diameter)^2^. A small portion of xenograft tumors were fixed overnight in paraformaldehyde followed by dehydration in graded ethanol and embedding in paraffin. Tissue sections were prepared for hematoxylin and eosin (H&E), SMS, Bim, Ki67, and activated caspase 3 staining. The remaining tumors were homogenized in 2% SDS lysis buffer and then processed for western blot analysis^63^.

### Bioinformatics and statistical analyses

Three datasets were used to determine the relative expression levels of *SMS* and *MYC* genes in human CRC patients. The Cancer Genome Atlas (TCGA) dataset, TCGA-COAD, contains 461 tumor samples and 41 matched normal samples. The Skrzypczak dataset contains 81 tumors and 24 normal samples. The Gaedcke dataset contains 65 matched pairs of tumor and normal samples. Expression of *SMS* and *MYC* in tumor versus normal samples was compared on the basis of two-tailed two-sample *t*-tests when all tumor and normal samples are from different individuals or linear mixed models when there are matched pairs of tumor and normal samples from the same individual. All statistical analyses for gene expression were performed using R (version 3.4.1).

Statistical analysis for each experiment were performed as described in the corresponding figure legends. Data between groups were compared using a two-tailed unpaired *t* test, one-way ANOVA test, *χ*^2^-test or Spearman’s rank-order correlation test. All data are presented as mean ± SEM. Differences between groups were considered statistically significant at *p* < 0.05. GraphPad Prism (version 8.1.1) software were used for these analyses. Unless otherwise stated, the experiments were performed at least three times with similar results. IHC staining and animal experiment were performed for a single time with biological replicates indicated in the figure legend and Methods section. Western blot and ChIP analyses were performed two times with similar results.

### Reporting summary

Further information on research design is available in the Nature Research Reporting Summary linked to this article.

## Supplementary information

Supplementary Information

Reporting Summary

## Data Availability

The TCGA Colon Adenocarcinoma (TCGA-COAD) mRNA-sequencing data referenced during the study are available in a public repository from the GDC Data Portal (https://portal.gdc.cancer.gov/projects/TCGA-COAD). The raw microarray RNA-sequencing data “Skrzypczak Colorectal” and “Gaedcke Colorectal” are available from Gene Expression Omnibus (https://www.ncbi.nlm.nih.gov/gds/) with accession GSE20916 and GSE20842, respectively. The corresponding processed and normalized data are available from Oncomine (https://www.oncomine.org/). The source data underlying Figs. 1b–d, f–h, 2a–c, e–k, 3a, b, d–j, 4, 5a, c–e, g–n, 6, and 7a–c, e–l and Supplementary Figs. 1a, b, 2a, b, d, 3b–d, 4a–c, e, f, 5–7, 8a–g, 9, 10b, and 11b–d are provided as a Source Data file. All the other data supporting the findings of this study are available within the article and its supplementary information files and from the corresponding author upon reasonable request. A reporting summary for this article is available as a Supplementary Information file.

## Source data

Source Data
