# Supplementary material for: Spermine synthase and MYC cooperate to maintain colorectal cancer cell survival by repressing Bim expression
Source: Nat Commun. 2020 Jun 26;11:3243. doi: 10.1038/s41467-020-17067-x (PMC7320137; doi:10.1038/s41467-020-17067-x)
Supplement: Supplementary file 1 — Supplementary Information [file 41467_2020_17067_MOESM1_ESM.pdf]

Supplementary Information

Spermine synthase and MYC cooperate to maintain colorectal cancer cell survival by repressing Bim expression

Guo et al.

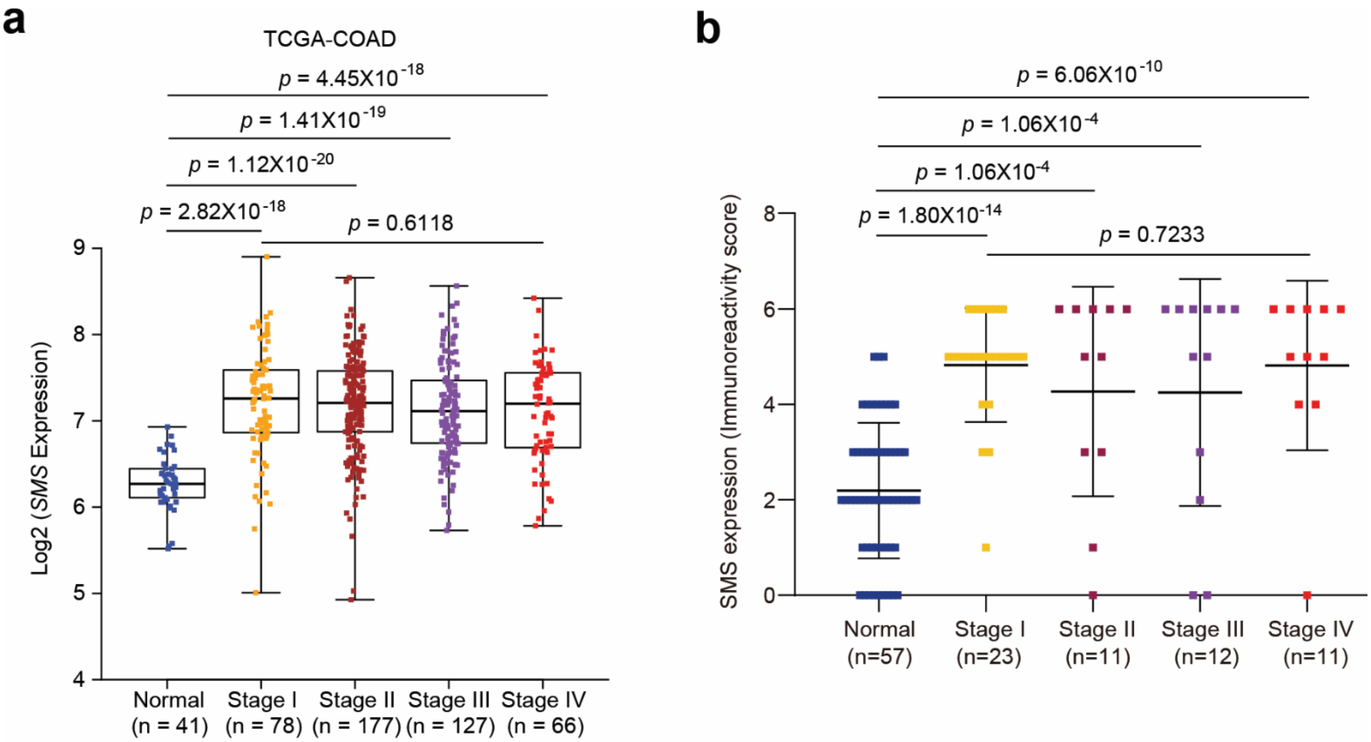

c

Patient ID	Gender	Age	Pathology	Staging
pt2893	M	51	Adenocarcinoma	T3, N0, Mx
pt2405	F	57	Mucinous adenocarcinoma	T4a, N1c, M1b
pt2769	F	56	Adenocarcinoma	T4a, N1b
pt2647	M	78	Adenocarcinoma	T3, N0
pt2751	M	82	Adenocarcinoma	T4a, N1c, M1a
pt2293	M	76	Adenocarcinoma	T3, N0

Supplementary Figure 1. SMS is upregulated in CRC patient samples. (**a**) The TCGA CRC dataset was used for the analyses of the stage-dependent expression of *SMS* mRNA in CRC patients. Five boxplots were created based upon the stage of cancer present (Normal, Stage I, Stage II, Stage III, and Stage IV). (**b**) SMS expression with the indicated immunoreactivity score from each stage of CRC patients and the adjacent normal control tissues was analyzed as shown in Fig. 1e, f. (**c**) De-identified patient data associated with tissues used for the analysis of SMS protein expression by western blot in Fig. 1g. Data are presented as mean values ± SEM in **b**. The indicated *p*-values in **a** and **b** were determined by linear mixed model followed by Holm method for multi-comparison adjustment to compare SMS expression across normal and different CRC stages, or by a one-way ANOVA test to compare SMS expression among the four CRC stages. Source data are provided as a Source Data file.

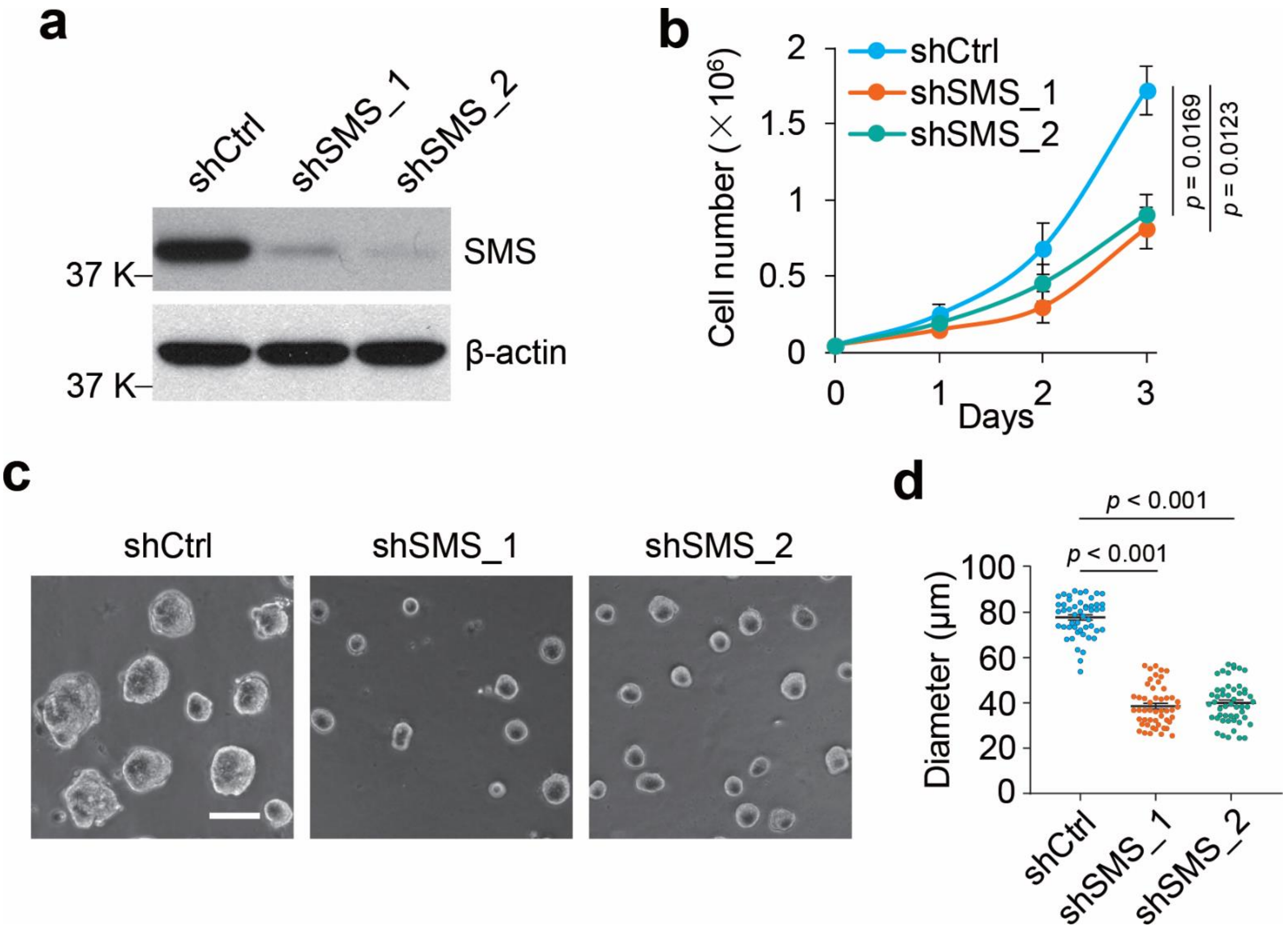

Supplementary Figure 2. Knockdown of SMS expression inhibits CRC cells growth. (**a**) HCT116 cells with stable expression of two different sets of SMS shRNAs (shSMS_1 and shSMS_2) or control shRNA (shCtrl) were analyzed by western blot for the indicated proteins. (**b**) HCT116 shCtrl or shSMS cells were assessed for cell growth over 3 days. (**c**) Representative phase-contrast images of HCT116 shCtrl or shSMS cells cultured in 3D Matrigel for 4 days. Scale bar, 100 µm. (**d**) The diameter of 50 randomly chosen spheroids as shown in (**c**) were measured. All graphic data are presented as mean ± SEM (n=3 independent experiments in **b**; n=50 spheroids in **d**). The indicated *p*-values were determined by two-tailed unpaired *t* test. Source data are provided as a Source Data file.

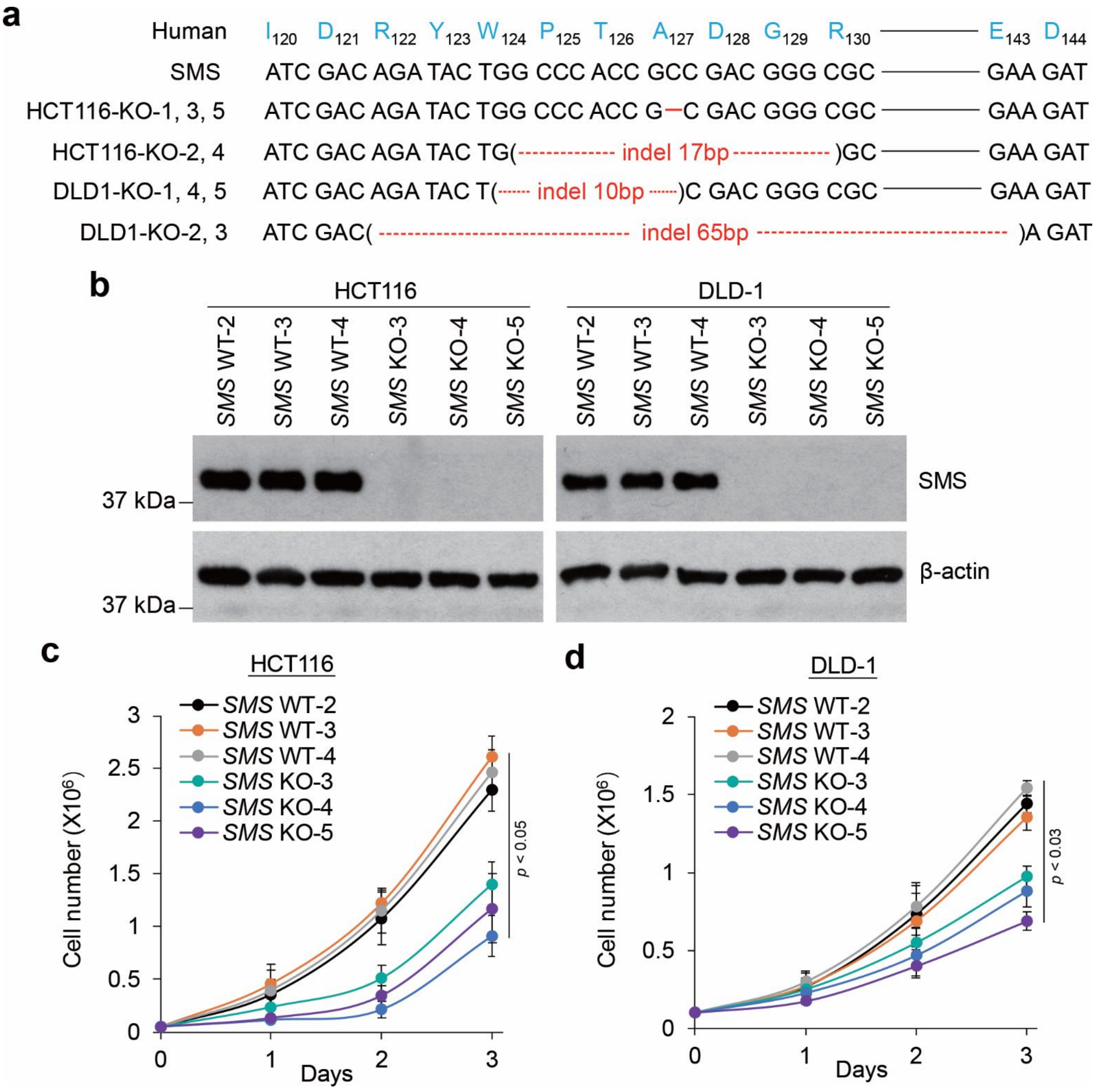

Supplementary Figure 3. Multiple *SMS*-knockout CRC cell clones demonstrate a slow growth rate. (**a**) Generation of human *SMS* knockout CRC cell lines. Exon 5 of human *SMS* was targeted by the CRISPR-Cas9 gRNA system. The sequences of internal deletion (indel) are shown in the five HCT116 or DLD-1 *SMS* knockout (KO) cell clones used in the study. Amino acid codes are indicated with blue characters. (**b**) Cells from three *SMS* knockout (KO) HCT116 or DLD-1 clones, and their control wild-type (WT) cells were analyzed by western blot for the indicated proteins. (**c, d**) *SMS*-WT or *SMS*-KO HCT116 (**c**) and DLD-1 (**d**) cells were assessed for cell growth over 3 days. Cell numbers are presented as mean ± SEM (n=3 independent experiments). The indicated *p*-values were determined by two-tailed unpaired *t* test. Source data are provided as a Source Data file.

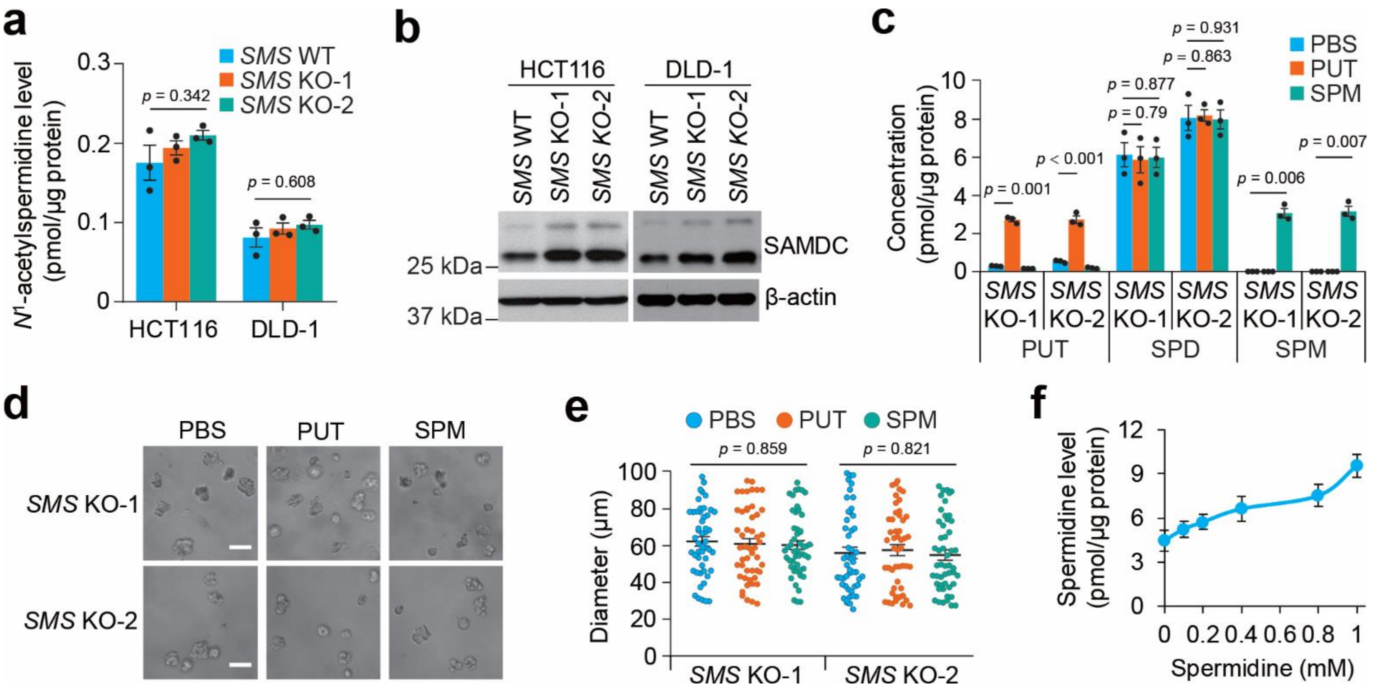

Supplementary Figure 4. Addition of exogenous putrescine and spermine does not reverse cell growth inhibition by *SMS* KO. (**a**) The levels of *N*[1]-acetylspermidine in *SMS*-WT or *SMS*-KO HCT116 cells were determined by LC-MS. (**b**) *SMS*-KO HCT116 or DLD-1 cells and their control WT cells were analyzed by western blot for the indicated proteins. (**c**) *SMS*-KO HCT116 cells were treated with 1 mM putrescine (PUT), 20 µM spermine (SPM), or with PBS as control for 24 h, followed by LC-MS analysis for the levels of PUT, spermidine (SPD) and SPM. (**d**) Representative phase-contrast images of *SMS*-KO HCT116 cells cultured in 3D Matrigel in the presence of 1 mM PUT, 20 µM spermine SPM, or PBS as control for 4 days. Scale bar, 100 µm. (**e**) The diameter of 50 randomly chosen spheroids as shown in (**d**) were measured. (**f**) HCT116 cells were treated with the indicated concentrations of spermidine for 24 h, followed by LC-MS analysis for the levels of spermidine. All graphic data are presented as mean ± SEM (n=3 independent experiments in **a**, **c** and **f**; n=50 spheroids in **e**). The indicated *p*-values were determined by one-way ANOVA test in **a** and **e**, or two-tailed unpaired *t* test in **c**. Source data are provided as a Source Data file.

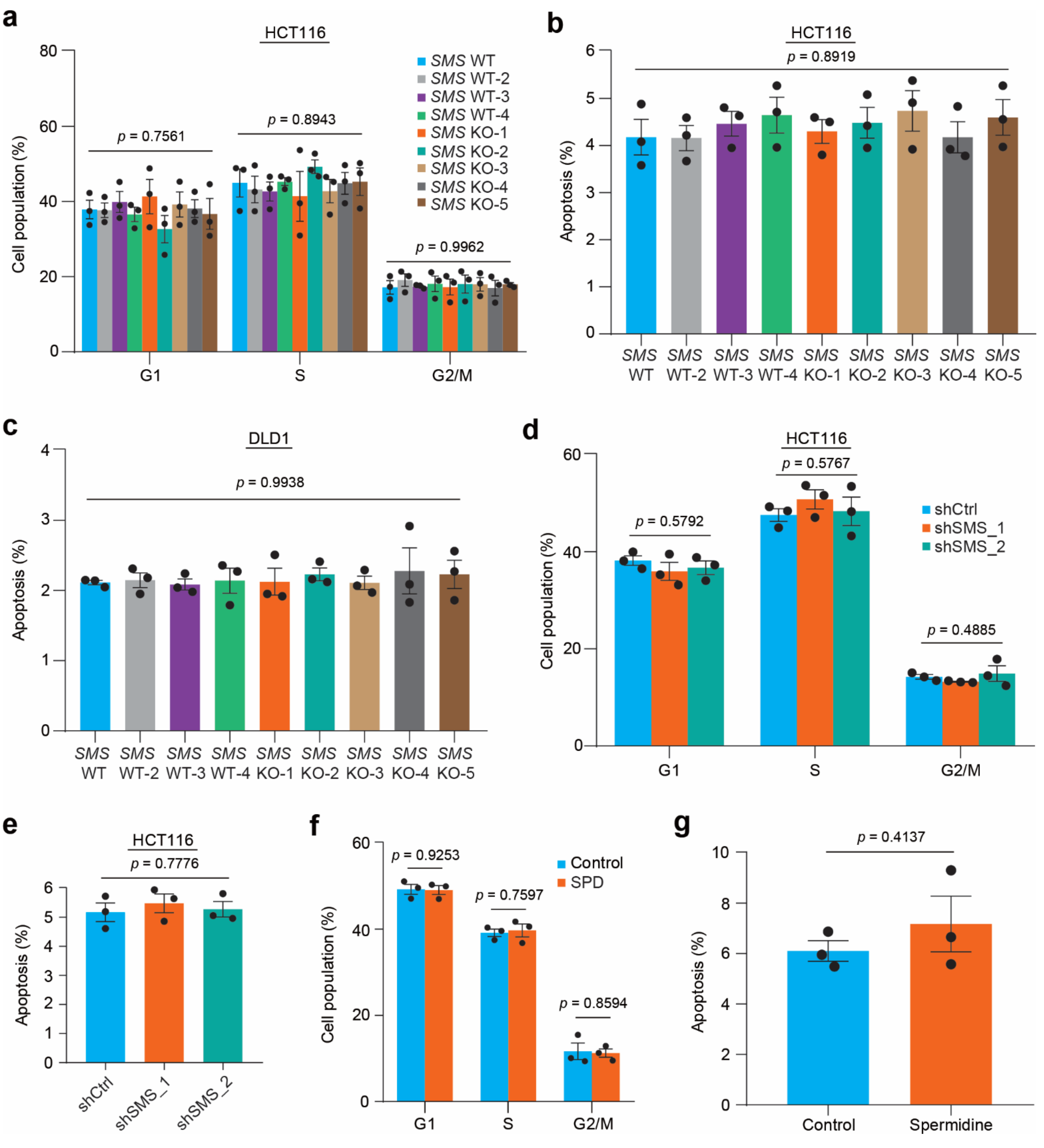

Supplementary Figure 5. SMS depletion or spermidine has no effect on cell cycle progression and survival in CRC cells. (**a-c**) *SMS*-WT or *SMS*-KO HCT116 or DLD-1 cells were assessed for the analyses of cell cycle (**a**) and apoptosis (**b, c**) using flow cytometry. (**d, e**) HCT116 cells with stable expression of two different sets of SMS shRNAs (shSMS_1 and shSMS_2) or control shRNA (shCtrl) were assessed for the analyses of cell cycle (**d**) and apoptosis (**e**) using flow cytometry. (**f, g**) HCT116 cells were exposed to 1 mM spermidine (SPD) for 72 h, followed by flow cytometry analyses of cell cycle (**f**) and apoptosis (**g**). All graphic data are presented as mean values ± SEM (n=3 independent experiments). The indicated *p*-values were determined by one-way ANOVA test in **a**-**e**, or two-tailed unpaired *t* test in **f** and **g**. Source data are provided as a Source Data file.

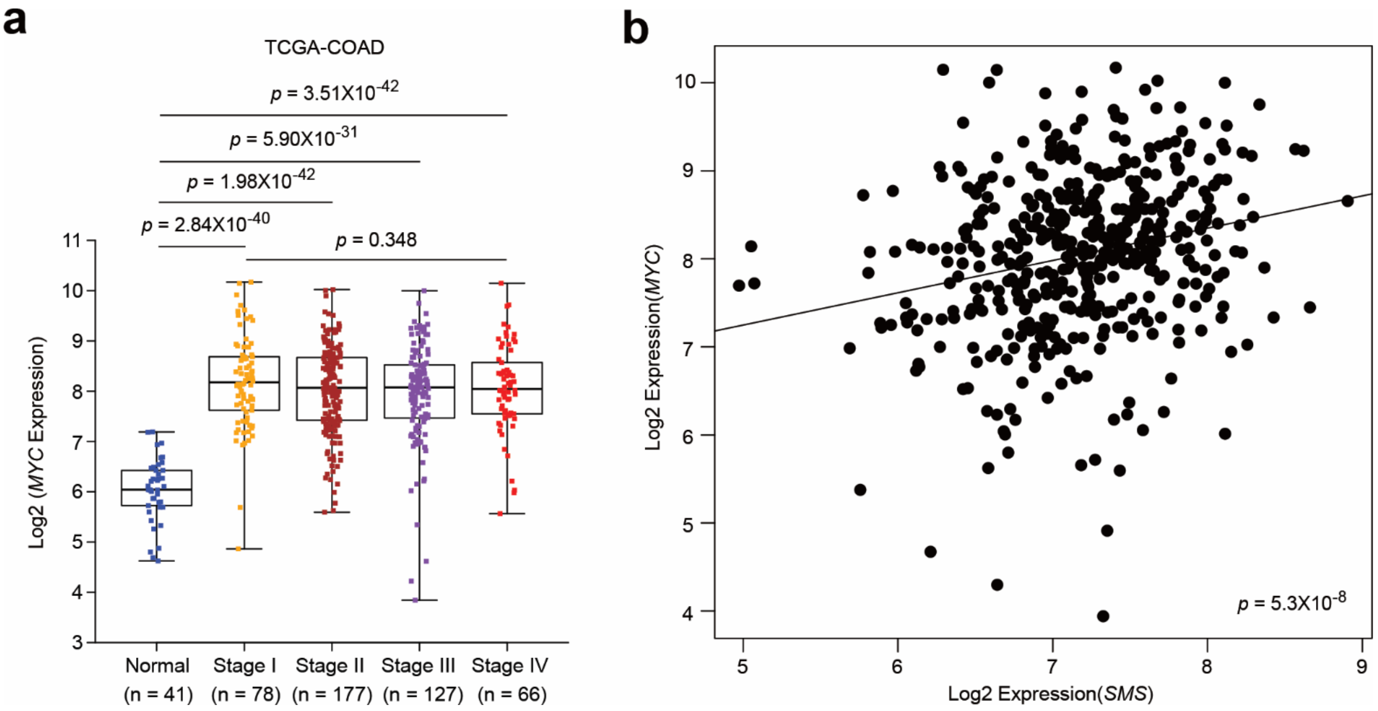

Supplementary Figure 6. MYC expression significantly correlates with SMS expression in CRC. (**a**) The TCGA CRC dataset was used for the analyses of the stage-dependent expression of *MYC* mRNA in CRC patients. Five boxplots were created based upon the stage of cancer present (Normal, Stage I, Stage II, Stage III, and Stage IV). The indicated *p*-values were determined by linear mixed model followed by Holm method for multi-comparison adjustment to compare *MYC* expression across normal and different CRC stages, or by a one-way ANOVA test to compare *MYC* expression among the four CRC stages. (**b**) The TCGA CRC dataset (n=461) was used for the analysis of the correlation between the expression of *MYC* and *SMS* mRNAs. Statistical significance of the correlation was determined by the Spearman's test with the *p*-value indicated. Source data are provided as a Source Data file.

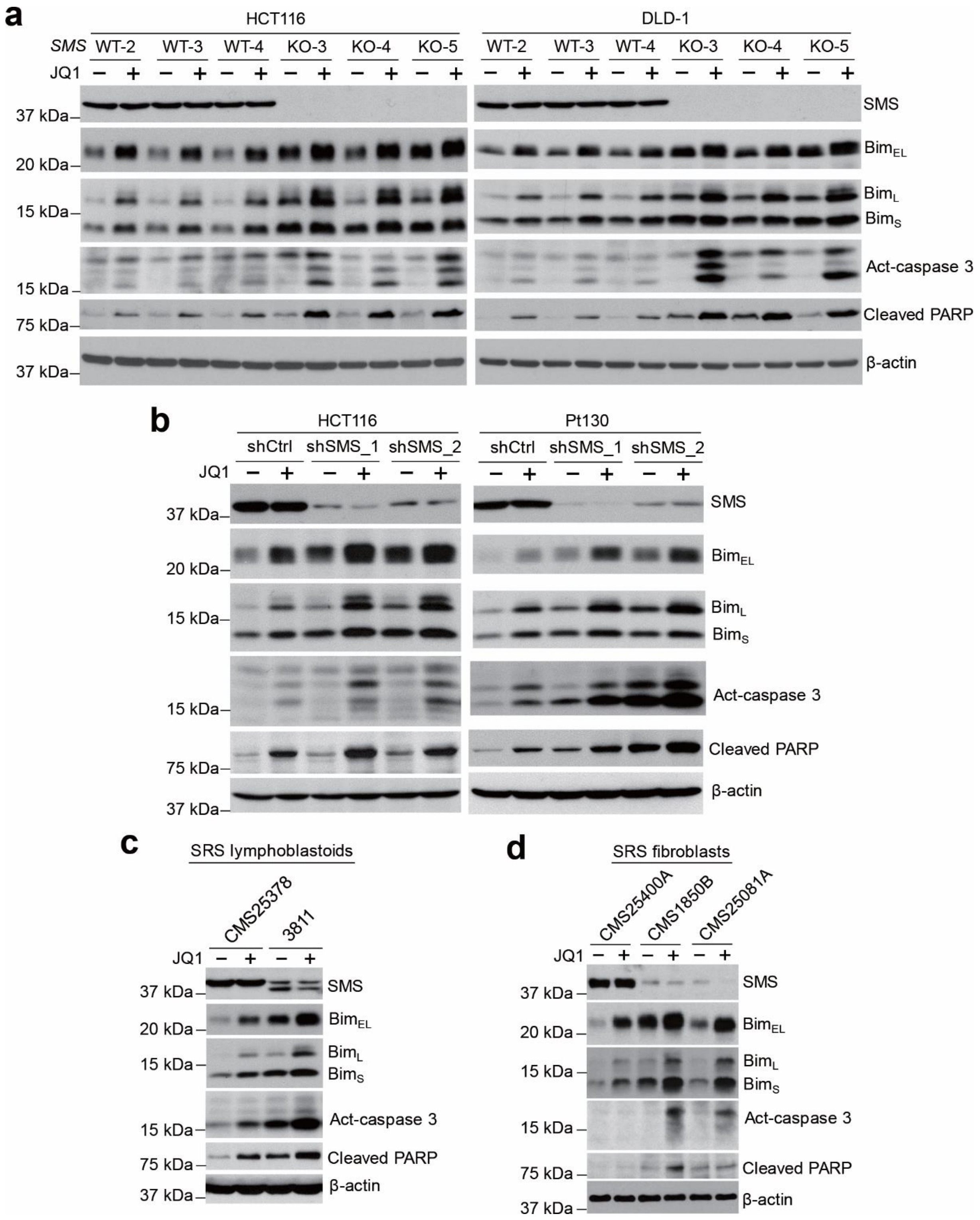

Supplementary Figure 7. SMS loss enhances JQ1-induced Bim expression and caspase 3 activation. (**a**) Cells from three *SMS*-KO HCT116 or DLD-1 clones, and their repective three control WT clones were treated with 1 µM JQ1 for 24 h, followed by western blot analysis for the indicated proteins. (**b**) HCT116 or Pt130 cells with stable expression of SMS shRNAs (shSMS_1 and shSMS_2) or control shRNA (shCtrl) were treated with 1 µM JQ1 for 24 h, followed by western blot analysis for the indicated proteins. (**c, d**) SRS lymphoblastoids or fibroblasts were treated with 1 µM JQ1 for 24 h, followed by western blot analysis for the indicated proteins. Source data are provided as a Source Data file.

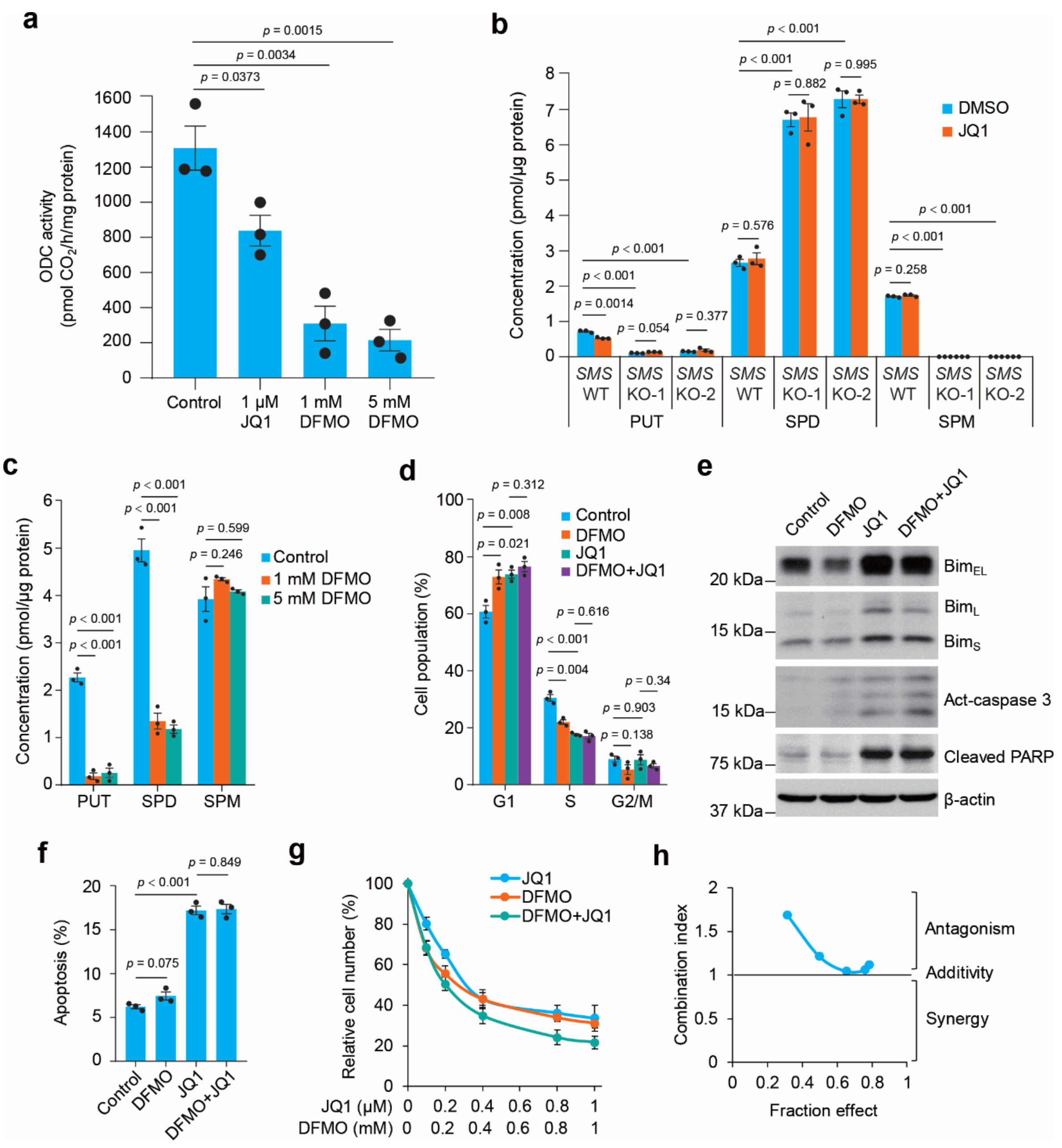

Supplementary Figure 8. The ODC inhibitor DFMO induces G1 accumulation but does not potentiate JQ1-induced apoptosis. (**a**) HCT116 cells were treated with 1 μM JQ1, 1 mM or 5 mM DFMO for 24 h, followed by assay for the ODC activity. (**b**) *SMS*-WT or *SMS*-KO HCT116 cells were treated with 1 μM JQ1 or with DMSO for 24 h, followed by LC-MS analysis for the levels of putrescine (PUT), spermidine (SPD) and spermine (SPM). (**c**) HCT116 cells were treated with 1 mM or 5 mM DFMO or with PBS as control for 24 h, followed by LC-MS analysis for the levels of PUT, SPD and SPM. (**d-f**) HCT116 cells were treated with 1 mM DFMO and 1 μM JQ1, alone or in combination for 24 h (**e**) or 72 h (**d, f**), followed by flow cytometry analyses of cell cycle (**d**) and apoptosis (**f**) or western blot analysis of the indicated proteins (**e**). (**g**) HCT116 cells were treated with JQ1 and DFMO, alone or in combination, with the indicated concentrations for 72 h. Results are expressed as a percentage of viable cell number relative to the value obtained in DMSO-treated control cells. (**h**) HCT116 cells were treated with a combination of JQ1 and DFMO. Combination index values were determined using the Chou-Talalay method for drug combinations with a fractional effect between 0.30 and 0.90 (30-90% of cell growth inhibition relative to control). Data are presented as mean values ± SEM (n=3 independent experiments) in **a**-**d**, **f** and **g**. The indicated *p*-values were determined by two-tailed unpaired *t* test. Source data are provided as a Source Data file.

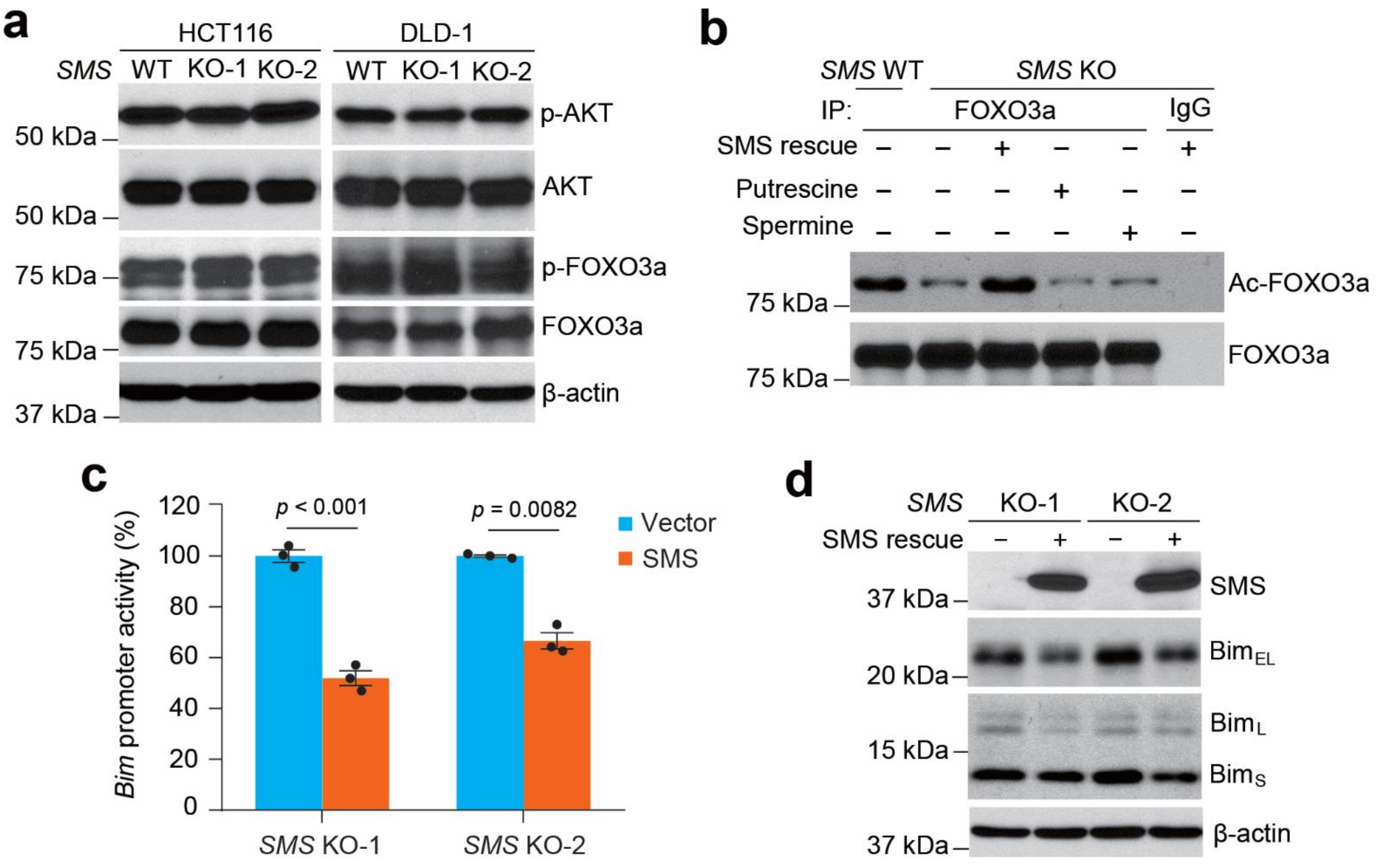

Supplementary Figure 9. Re-expression of SMS represses Bim expression in *SMS*-KO cells. (**a**) Two *SMS*-KO HCT116 or DLD-1 clones and their control WT cells were analyzed by western blot for the indicated proteins. (**b**) *SMS*-WT HCT116 cells, and *SMS*-KO HCT116 cells re-expressing SMS or treated with 1 mM putrescine or 20 μM spermine for 24 h, were lysed and immunoprecipitated with FOXO3a antibody or IgG as control, followed by western blot analysis for the indicated proteins. (**c, d**) *SMS*-KO HCT116 cells with re-expression of SMS or vector control were subjected to analysis of *Bim* promoter activity (**c**), or western blot analysis for the indicated proteins (**d**). The graphic data in **c** are presented as mean values ± SEM (n = 3 independent experiments). The indicated *p*-values were determined by two-tailed unpaired *t* test. Source data are provided as a Source Data file.

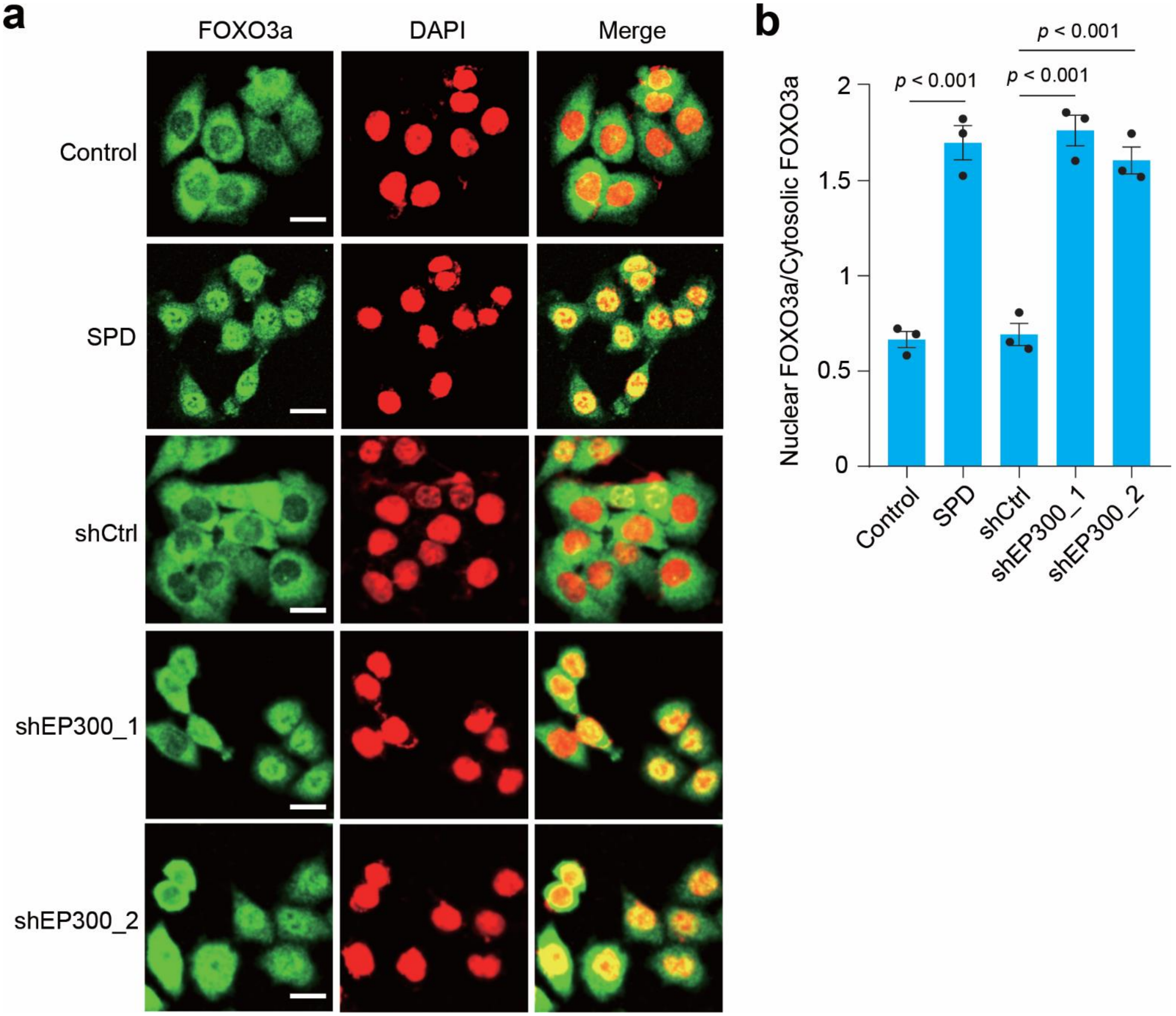

Supplementary Figure 10. FOXO3a is translocated from the cytoplasm to nucleus by spermidine treatment or EP300 knockdown. (**a**) HCT116 cells treated with 1 mM spermidine (SPD) or with PBS as control for 24 h, and HCT116 cells with stable expression of two different sets of EP300 shRNAs or control shRNA, were stained for FOXO3a (green) and DAPI (red). (**b**) The distribution of FOXO3a in the cytosol and nucleus shown in (**a**) was analyzed using Image J. n = 30 cells per condition. Scale bar, 25 µm. Data are presented as mean values ± SEM (n = 3 independent experiments). The indicated p-values were determined by two-tailed unpaired *t* test. Source data are provided as a Source Data file.

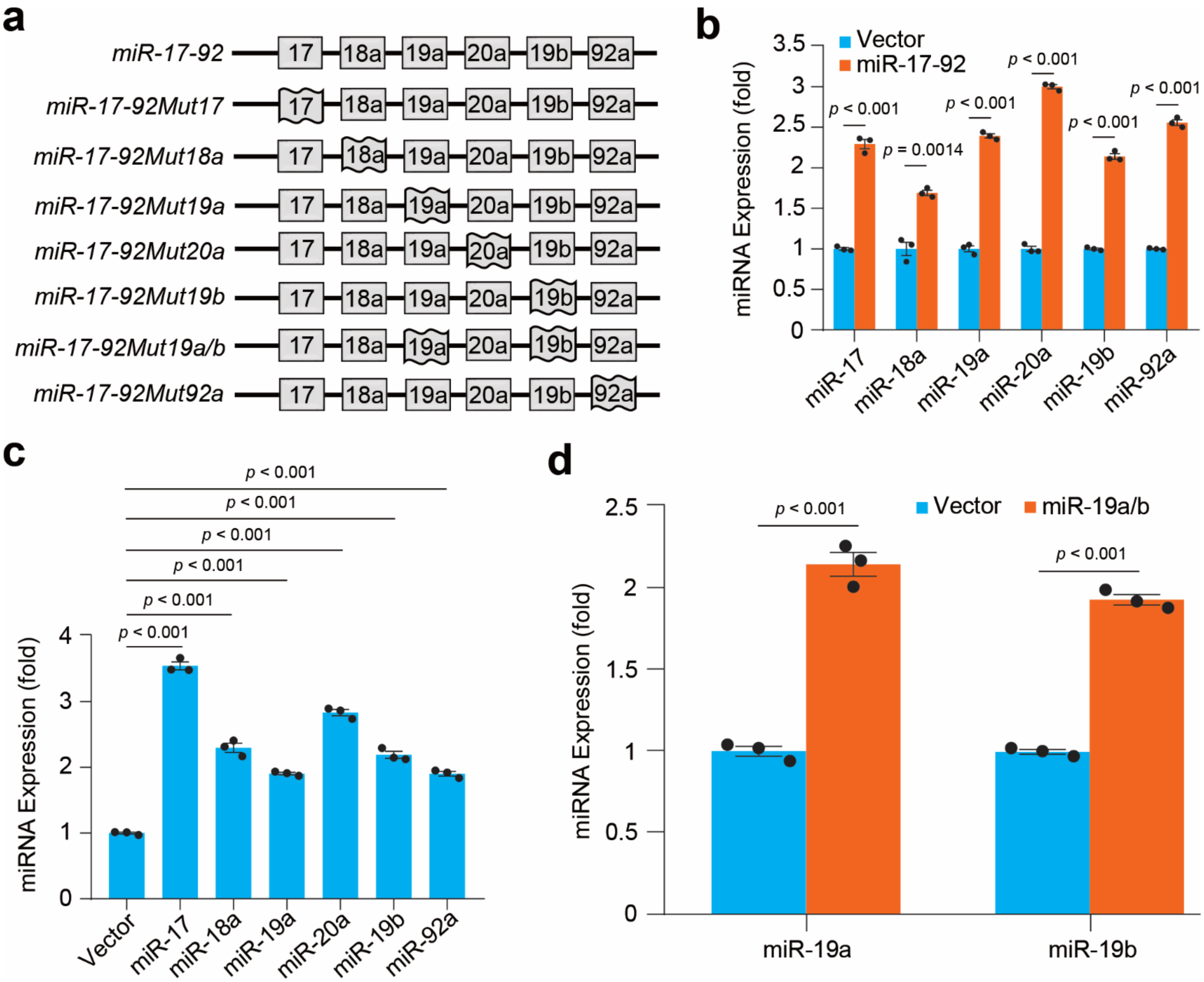

Supplementary Figure 11. RT-PCR analysis on the expression of the individual miR-17-92 members. (**a**) The gene structure of the *miR-17-92* polycistron and its mutated derivaties. (**b**) The expression levels of the indicated miRNAs were quantified by RT-PCR analysis in HCT116 cells with stable expression of the miR-17-92 cluster or vector control. (**c**) The expression levels of the individial miRNAs were quantified by RT-PCR analysis in HCT116 cells with stable expression of miR-17, miR-18a, miR-19a, miR-20a, miR19b, miR-92a or vector control. (**d**) The expression levels of miR-19a and miR-19b were quantified by RT-PCR analysis in HCT116 cells with stable expression of miR-19a/b or vector control. All graphic data are presented as mean values ± SEM (n = 3 independent experiments). The indicated p-values were determined by two-tailed unpaired *t* test. Source data are provided as a Source Data file.

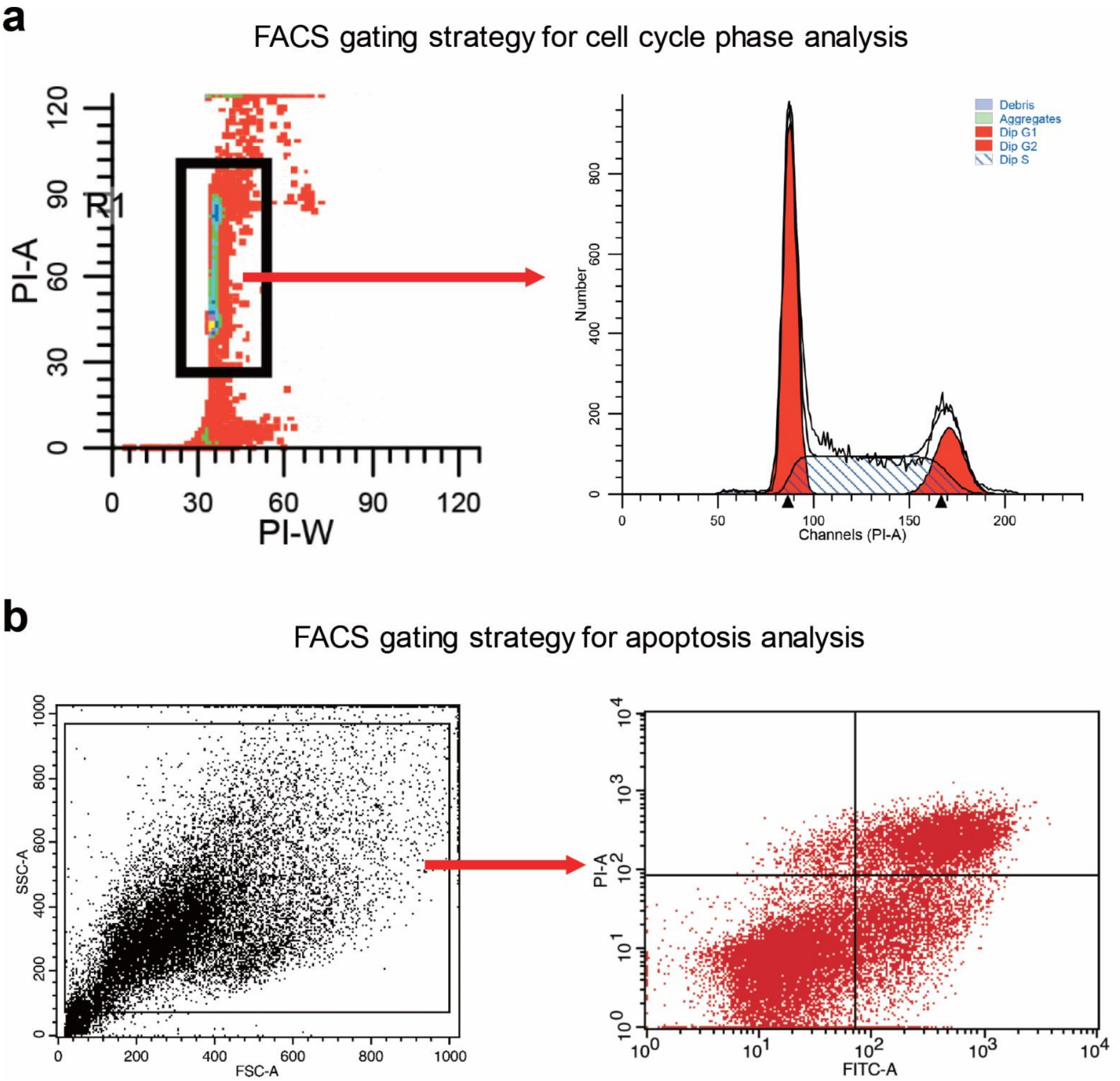

Supplementary Figure 12. FACS gating strategy for cell cycle and apoptosis analyses. (**a**) Representative gating strategy on single cell population as determined by a PI width (PI-W) vs. PI area (PI-A) dot plot for cell cycle analysis. (**b**) Representative gating strategy for excluding debris in flow cytometry analysis of apoptosis. Small debris were excluded on a Side-Scatter (Area) vs. Forward-Scatter (Area) dot plot. Then, the cell population was analyzed by 2 fluorescence channels including FITC and PI. The boundaries between "positive" and "negative" staining cells were gated according to the control groups.

Supplementary Table 1. Primers for construction of human *SMS* gene, *Bim* promoter, *Bim* 3'UTR, *Bim* 3'UTR Mut 19, mir-17, mir-19a and mir-17-92 mutants

Constructs	Forward Primers (5'-3')	Reverse Primers (5'-3')
SMS	CGGGATCCATGGCAGCAGCACGGCAC	ACGCGTCGACTCAGGGTTTAGCTTTCTTCCAAA
Bim promoter	GGGGTACCACATGCCTCCCGCCCTCA	CTAGCCATGGGAGCTCCAACAAACTGCAGACC
Bim 3'UTR WT	GCTCTAGAATGTATTTGGATCTGGGCAC	GGAATTCCATATGACTAGTTGAAAGTTTTATTT
Bim 3'UTR Mut19	AATTACCCTGTCAAAATTCATCAGTTACACACACAAGTAAGCCAGGGAAACTGCAATACAAA	TTTGTATTGCAGTTTCCCTGGCTTACTTGTGTGTGTAACTGATGAATTTTGACAGGGTAATT
MSCV-miR-17	CCGCTCGAGTTGAGGTGTTAATTCTAATTATC	GGAATTCGCACCTTAGAACAAAAAGCA
MSCV-miR-19a	CCGCTCGAGAGCATCTACTGCCCTAAGTGC	GGAATTCTCTACAGCTGGCAGTACTTTAA
MSCV-miR-17-92Mut17	CCAGTCAGAATAATGTCAAtccacaataatcGCAGGTAGTGATATGTGCATC	GATGCACATATCACTACCTGCGATTATTGTGGATTGACATTATTCTGACTGG
MSCV-miR-17-92Mut18a	GATGTTGAGTGCTTTTTGTTgctcacacatcaTAGTGCAGATAGTGAAGTAG	CTACTTCACTATCTGCACTATGATGTGTGAGCAACAAAAAGCACTCAACATC
MSCV-miR-17-92Mut19a	CTACAAGAAGAATGTAGTTGacatttaactacCAAAACTGATGGTGGCCTGC	GCAGGCCACCATCAGTTTTGGTAGTTAAATGTCAACTACATTCTTCTTGTAG
MSCV-miR-17-92Mut19b	CTGTGTGATATTCTGCTGacatttaagtacCAAAACTGACTGTGGTAGTG	CACTACCACAGTCAGTTTTGGTACTTAAATGTCAGCAGAATATCACACAG
MSCV-miR-17-92Mut20a	GACAGCTTCTGTAGCACTAAtaaacaataatcGCAGGTAGTGTTTAGTTATC	GATAACTAAACACTACCTGCGATTATTGTTTATTAGTGCTACAGAAGCTGTC
MSCV-miR-17-92Mut92a	CAATGCTGTGTTTCTGTATGGTtaacattaacatCCGGCCTGTTGAGTTTG	CAAACTCAACAGGCCGGATGTTAATGTTAACCATACAGAAACACAGCATTG

Supplementary Table 2. shRNA sequences for targeting the indicated human genes

Constructs	Target sequences (5'-3')
SMS shRNA_1	CCTCTCAATGAAAGTGTTGAA
SMS shRNA_2	GCGATGTCTTAGACAATCTTA
Bim shRNA_1	ACGAATGGTTATCTTACGACT
Bim shRNA_2	AGCCGAAGACCACCCACGAAT
PUMA shRNA_1	GAGGGTCCTGTACAATCTCAT
PUMA shRNA_2	GCAAATGAGCCAAACGTGACC
EP300 shRNA_1	CGGAAACAGTGGCACGAAGAT
EP300 shRNA_2	GCGGAATACTACCACCTTCTA
MYC shRNA_1	CCTGAGACAGATCAGCAACAA
MYC shRNA_2	CAGGAACTATGACCTCGACTA
BRD4 shRNA_1	GAGGAAGAGGACAAGTGCAAG
BRD4 shRNA_2	AAGAAGGGAGTGAAGAGGAAA

Supplementary Table 3. Antibodies used for immunofluorescence (IF), western blot (WB), immunoprecipitation (IP), ChIP and IHC analyses

Antibody	Usage/Dilution	Species	Provider	Catalog #
Anti-SMS	WB: 1:1000<br>IF: 1:250<br>IHC: 1:200	Rabbit monoclonal	Abcam	ab156879
Anti-Ki67	IHC: 1:5000	Rabbit polyclonal	Abcam	ab15580
Anti-PUMA	WB: 1:1000	Rabbit polyclonal	Abcam	ab9643
Anti-Cleaved Caspase-3	WB: 1:500<br>IHC: 1:5000	Rabbit monoclonal	Cell Signaling Technology	9664
Anti-MYC	WB: 1:1000	Rabbit monoclonal	Cell Signaling Technology	5605
Anti-AKT	WB: 1:1000	Rabbit polyclonal	Cell Signaling Technology	9272
Anti-Phospho-AKT	WB: 1:1000	Rabbit monoclonal	Cell Signaling Technology	4060
Anti-ERK	WB: 1:1000	Rabbit polyclonal	Cell Signaling Technology	9102
Anti-Phospho-ERK	WB: 1:1000	Rabbit polyclonal	Cell Signaling Technology	9101
Anti-Cleaved PARP	WB: 1:1000	Rabbit polyclonal	Cell Signaling Technology	9541
Anti-Bcl-xL	WB: 1:1000	Rabbit monoclonal	Cell Signaling Technology	2764
Anti-Mcl-1	WB: 1:1000	Rabbit polyclonal	Cell Signaling Technology	4572
Anti-XIAP	WB: 1:500	Rabbit monoclonal	Cell Signaling Technology	2045
Anti-Noxa	WB: 1:1000	Rabbit monoclonal	Cell Signaling Technology	14766
Anti-Bad	WB: 1:1000	Rabbit monoclonal	Cell Signaling Technology	9239
Anti-BID	WB: 1:1000	Rabbit polyclonal	Cell Signaling Technology	2002
Anti-Bim	WB: 1:1000<br>IHC: 1:200	Rabbit monoclonal	Cell Signaling Technology	2933
Anti-Acetylated-Lysine	WB: 1:1000	Rabbit polyclonal	Cell Signaling Technology	9441
Anti-Phospho-FOXO3a	WB: 1:1000	Rabbit polyclonal	Cell Signaling Technology	9464
Anti-BRD4	WB: 1:1000	Rabbit monoclonal	Cell Signaling Technology	13440
Anti-HA-Tag	WB: 1:1000	Mouse monoclonal	Cell Signaling Technology	2367
Anti-FOXO3a	WB: 1:1000<br>IP: 1:50	Rabbit monoclonal	Cell Signaling Technology	12829
Anti-FOXO3a	IF:1:800	Mouse monoclonal	Cell Signaling Technology	99199
Anti-FOXO3a	ChIP: 1:100	Mouse monoclonal	Santa Cruz Biotechnology	sc-48348
Anti-Bcl-2	WB: 1:250	Mouse monoclonal	Santa Cruz Biotechnology	sc-7382
Anti-Bax	WB: 1:250	Rabbit polyclonal	Santa Cruz Biotechnology	sc-526
Anti-EP300	WB: 1:200	Mouse monoclonal	Santa Cruz Biotechnology	sc-32244
Anti-Survivin	WB: 1:1000	Rabbit polyclonal	Novus Biologicals	NB500-201
Anti-AMD1 (SAMDC)	WB: 1:1000	Rabbit polyclonal	Proteintech	11052-1-AP
Anti-β-actin	WB: 1:10000	Mouse monoclonal	Sigma-Aldrich	A5441
Anti-mouse IgG with TRITC	IF: 1:500	Goat polyclonal	Jackson ImmunoResearch Laboratories	111-025-166
Anti-rabbit IgG with Alexa Fluor 680	IF: 1:500	Donkey polyclonal	Jackson ImmunoResearch Laboratories	711-625-152
