# Supplementary material for: Spermine synthase and MYC cooperate to maintain colorectal cancer cell survival by repressing Bim expression
Source: Nat Commun. 2020 Jun 26;11:3243. doi: 10.1038/s41467-020-17067-x (PMC7320137; doi:10.1038/s41467-020-17067-x)
Supplement: Supplementary file 2 — Reporting Summary [file 41467_2020_17067_MOESM2_ESM.pdf]

nature research

Corresponding author(s): Qing-Bai She, Side Liu

Last updated by author(s): May 29, 2020

Reporting Summary

Nature Research wishes to improve the reproducibility of the work that we publish. This form provides structure for consistency and transparency in reporting. For further information on Nature Research policies, see Authors & Referees and the Editorial Policy Checklist.

Statistics

For all statistical analyses, confirm that the following items are present in the figure legend, table legend, main text, or Methods section.

n/a	Confirmed	
☐	☒	The exact sample size (n) for each experimental group/condition, given as a discrete number and unit of measurement
☐	☒	A statement on whether measurements were taken from distinct samples or whether the same sample was measured repeatedly
☐	☒	The statistical test(s) used AND whether they are one- or two-sided<br>*Only common tests should be described solely by name; describe more complex techniques in the Methods section.*
☐	☒	A description of all covariates tested
☐	☒	A description of any assumptions or corrections, such as tests of normality and adjustment for multiple comparisons
☐	☒	A full description of the statistical parameters including central tendency (e.g. means) or other basic estimates (e.g. regression coefficient) AND variation (e.g. standard deviation) or associated estimates of uncertainty (e.g. confidence intervals)
☐	☒	For null hypothesis testing, the test statistic (e.g. F, t, r) with confidence intervals, effect sizes, degrees of freedom and P value noted<br>*Give P values as exact values whenever suitable.*
☒	☐	For Bayesian analysis, information on the choice of priors and Markov chain Monte Carlo settings
☒	☐	For hierarchical and complex designs, identification of the appropriate level for tests and full reporting of outcomes
☒	☐	Estimates of effect sizes (e.g. Cohen's d, Pearson's r), indicating how they were calculated

Our web collection on statistics for biologists contains articles on many of the points above.

Software and code

Policy information about availability of computer code

Data collection	Cell counts were collected using the Vi-CELL XR 2.06 (Beckman Coulter). Images were acquired by the Nikon Eclipse Ti-E inverted microscope for live imaging or Nikon A1+-Ti2 confocal microscope for immunofluorescence. Flow cytometry data were acquired on a LSR II flow cytometer (BD Biosciences). RT-PCR data were collected using a StepOne RT-PCR system (Applied Biosystems). Polyamine data were acquired by an Ultimate 3000 ultra-high performance liquid chromatography and a Q-exactive mass spectrometer (Thermo Fisher Scientific). All publicly available data referenced in our manuscript have been collected from TCGA (https://portal.gdc.cancer.gov/projects/TCGA-COAD), GEO (https://www.ncbi.nlm.nih.gov/gds/), or the indicated websites.
Data analysis	Statistical analyses were performed using GraphPad Prism (v8.1.1) or R (v3.4.1). Flow cytometry data were analyzed using ModFit (v3.3) or Cell Quest Pro (v5.2.1). The cell sphere sizes were measured using Nikon NIS-Elements AR software (v5.00.00). The combination index data were analyzed using CompuSyn software (v1.0.1). Western blot and the distribution of FOXO3a in the cytosol and nucleus were quantified using Image J (v1.8.0). The guide RNAs were designed using the CRISPR designing tool (https://zlab.bio/guide-design-resources).

For manuscripts utilizing custom algorithms or software that are central to the research but not yet described in published literature, software must be made available to editors/reviewers. We strongly encourage code deposition in a community repository (e.g. GitHub). See the Nature Research guidelines for submitting code & software for further information.

Data

Policy information about availability of data

All manuscripts must include a data availability statement. This statement should provide the following information, where applicable:

- Accession codes, unique identifiers, or web links for publicly available datasets
- A list of figures that have associated raw data
- A description of any restrictions on data availability

The TCGA Colon Adenocarcinoma (TCGA-COAD) mRNA-sequencing data referenced during the study are available in a public repository from the GDC Data Portal (https://portal.gdc.cancer.gov/projects/TCGA-COAD). The raw microarray RNA-sequencing data “Skrzypczak Colorectal” and “Gaedcke Colorectal” are available

from Gene Expression Omnibus (https://www.ncbi.nlm.nih.gov/gds/) with accession GSE20916 and GSE20842, respectively. The corresponding processed and normalized data are available from Oncomine (https://www.oncomine.org/). The source data underlying Figs. 1b-d, f-h, 2a-c, e-k, 3a, b, d-j, 4, 5a, c-e, g-n, 6, and 7a-c, e-l and Supplementary Figs. 1a, b, 2a, b, d, 3b-d, 4a-c, e, f, 5-7, 8a-g, 9, 10b and 11b-d are provided as a Source Data file. All the other data supporting the findings of this study are available within the article and its supplementary information files and from the corresponding author upon reasonable request.

Field-specific reporting

Please select the one below that is the best fit for your research. If you are not sure, read the appropriate sections before making your selection.

☒ Life sciences ☐ Behavioural & social sciences ☐ Ecological, evolutionary & environmental sciences

For a reference copy of the document with all sections, see nature.com/documents/nr-reporting-summary-flat.pdf

Life sciences study design

All studies must disclose on these points even when the disclosure is negative.

Sample size	No statistical method was used to predetermine sample size. Sample size was chosen based on our prior studies using the same types of assays, as well as published literature, to ensure statistically significant results. In vitro studies used a standard n=3. In vivo studies were performed with 6 mice per treatment group with one tumor each, which we estimated would account for variability in tumor growth (Wang et al. Nature Communications 2017 and Huang et al. Nature Communications 2019).
Data exclusions	No data were excluded from the analyses.
Replication	Cell viabilty, luciferase reporter, RT-PCR, flow cytometry, immunofluorescence, polyamine, and ODC activity assays were reproduced in three independent experiments. All attempts at replication were successful. Animal studies and IHC analyses of colorectal cancer tissue microarrays were performed for a single time with biological replicates indicated in the figure legend and Methods section due to time and cost limitations.
Randomization	Mice were randomized to treatment groups after they developed palpable tumors. Colorectal cancer patients' samples were randomly allocated into different groups for IHC analysis. Randomization was not relevant for other experiments, as they were performed in cell lines.
Blinding	The experiments were not blinded, except the IHC staining analysis (staining and scoring) that was done in a blinded fashion.

Reporting for specific materials, systems and methods

We require information from authors about some types of materials, experimental systems and methods used in many studies. Here, indicate whether each material, system or method listed is relevant to your study. If you are not sure if a list item applies to your research, read the appropriate section before selecting a response.

Materials & experimental systems

n/a	Involved in the study	
☐	☒	Antibodies
☐	☒	Eukaryotic cell lines
☒	☐	Palaeontology
☐	☒	Animals and other organisms
☐	☒	Human research participants
☒	☐	Clinical data

Methods

n/a	Involved in the study	
☒	☐	ChIP-seq
☐	☒	Flow cytometry
☒	☐	MRI-based neuroimaging

Antibodies

Antibodies used	All the antibodies used in this study have been described in detail in Supplementary Table 3.
Validation	Antibodies used from Cell Signaling Technology (Supplementary Table 3) were validated by the supplier (https://www.cellsignal.com/contents/our-approach/cst-antibody-validation-principles/ourapproach-validation-principles) as described below.<br><br>"At Cell Signaling Technology (CST), we understand that there is no single assay that can determine the validity of an antibody. Confirming that an immunoreagent is sufficiently specific and sensitive depends on the application and protocol being used, the type and quality of sample being analyzed, and the inherent biophysical properties of the antibody itself.<br><br>To ensure our antibodies will work in your experiment, we adhere to the Hallmarks of Antibody Validation™, six complementary strategies that can be used to determine the functionality, specificity, and sensitivity of an antibody in any given assay. CST adapted the work by Uhlen, et. al., ("A Proposal for Validation of Antibodies." Nature Methods (2016)) to build the Hallmarks of Antibody Validation, based on our decades of experience as an antibody manufacturer and our dedication to reproducible science."

Anti-SMS antibody was validated using CRISPR KO cell lines. Anti-PUMA, Bcl-2, Bax, FOXO3a, EP300, survivin, AMD1 and b-actin antibodies were validated on cell lines and/or cell-line derived xenografts with shRNA-mediated knockdown of the targeted protein and/or known expression of the marker.

Eukaryotic cell lines

Policy information about cell lines

Cell line source(s)	HCT116, DLD-1, HCT15 and SW480 cells were obtained from ATCC. Pt130 primary colon cancer cells were provided by co-author, Yekaterina Zaytseva (University of Kentucky). SRS lymphoblastoid (3811) and fibroblast (CMS1850B, CMS25081A) cells and their control cells (CMS25378, CMS2400A) were provided by co-author, Charles Schwartz (Greenwood Genetic Center).
Authentication	All cell lines were authenticated using the short tandem repeat (STR) profiling (Genetica).
Mycoplasma contamination	All cell lines were periodically tested for mycoplasma contamination using e-Myco Plus kit (iNtRON Biotechnology). None of the cell lines were contaminated.
Commonly misidentified lines (See ICLAC register)	No commonly misidentified cell lines were used in this study.

Animals and other organisms

Policy information about studies involving animals; ARRIVE guidelines recommended for reporting animal research

Laboratory animals	NCr Nude mice (Taconic), males and females, 6 weeks old
Wild animals	No wild animals were used in this study.
Field-collected samples	No field-collected samples were used in this study.
Ethics oversight	All studies involving animals were performed under a protocol approved by the University of Kentucky Institutional Animal Care and Use Committee.

Note that full information on the approval of the study protocol must also be provided in the manuscript.

Human research participants

Policy information about studies involving human research participants

Population characteristics	Six pairs of primary colon tumors and adjacent normal control tissues collected from patients were used for western blot analysis. The clinical information of six patients is shown in Supplementary Fig. 1c.
Recruitment	All samples were obtained from the tissue bank in the Biospecimen Procurement and Translational Pathology Shared Resource Facility at the University of Kentucky Markey Cancer Center. No selection bias was used for this study.
Ethics oversight	All samples were collected with informed consent, and the study was performed under a protocol approved by the University of Kentucky Institutional Review Board.

Note that full information on the approval of the study protocol must also be provided in the manuscript.

Flow Cytometry

Plots

Confirm that:

- [x] The axis labels state the marker and fluorochrome used (e.g. CD4-FITC).
- [x] The axis scales are clearly visible. Include numbers along axes only for bottom left plot of group (a 'group' is an analysis of identical markers).
- [x] All plots are contour plots with outliers or pseudocolor plots.
- [x] A numerical value for number of cells or percentage (with statistics) is provided.

Methodology

Sample preparation	Sample preparation is described in the Methods.
Instrument	BD LSR II flow cytometer
Software	ModFit (v3.3) and Cell Quest Pro (v5.2.1) softwares were used to analyze cell cycle and apoptosis, respectively.

Cell population abundance	No cell sorting was performed. All flow cytometry data regard only sample analysis.
Gating strategy	Gating strategy is provided in Supplementary Figure 12.

☒ Tick this box to confirm that a figure exemplifying the gating strategy is provided in the Supplementary Information.
